# Supplementary material for: Characterization of a male specific region containing a candidate sex determining gene in Atlantic cod
Source: Sci Rep. 2019 Jan 15;9:116. doi: 10.1038/s41598-018-36748-8 (PMC6333804; doi:10.1038/s41598-018-36748-8)
Supplement: Supplementary file 1 — Supplementary Information [file 41598_2018_36748_MOESM1_ESM.pdf]

Supplementary Information

Characterization of a male specific region containing a candidate sex determining gene in Atlantic cod

Tina Graceline Kirubakaran[1], Øivind Andersen[1,2], Maria Cristina De Rosa[3], Terese Andersstuen[1], Kristina Hallan[1], Matthew Peter Kent[1*], Sigbjørn Lien[1*]

[1] Centre for Integrative Genetics (CIGENE), Department of Animal and Aquaculture Sciences (IHA), Faculty of Life Sciences (BIOVIT), Norwegian University of Life Sciences (NMBU), PO Box 5003, N-1430 Ås, Norway.

[2] Nofima, PO Box 5010, N-1430 Ås, Norway.

[3] Institute of Chemistry of Molecular Recognition - CNR c/o Institute of Biochemistry and Clinical Biochemistry, Catholic University of Rome, 00168 Rome, Italy

*Corresponding authors and shared last authorship

Sigbjørn Lien sigbjorn.lien@nmbu.no (SL)

Matthew Peter Kent matthew.peter.kent@nmbu.no (MPK)

Supplementary file 1: The position and the flanking sequences of the nine sex-specific SNPs on LG11 that is heterozygous in all 49 males and homozygous in all 53 females of Atlantic cod.

>LG11_12508441
TACAGGACACTTTATTCGCAGAGAGCCCCCTCAACAGACGCGTTTGCCTGAAGATCCAAAATCTCTAGGGTTAAAGTCTTGAAT
ACCAGAACAAGTGAGG[G/T]AAAGTTCTACCTAGACTGAAAGGATTTGTCCTCGTTTCTAAATGCATTCATTGCTACCCTGCTGG
ATCTGTGGACGTCTTTTCGAGATGGAAGACTGCTA

>LG11_12509561
TGACTCCTCCTTTTGATACTTCCTGACACAATTTGACACAATCAGCTCGTACAGTTTCAGTTGTGGTTTACATATGGGTTGAAGC
TGTGATCTCAGCACC[T/A]TCACCATGAAGTAAATAAAGTAATCACCCAAATTACTAAATGTAATGACCTGAGTTTTACACCAGA
TGGTGATGTCGCTGCATTCTGTGGATCACTTCAT

>LG11_12519007
TCAACGCACAACGTGTGCCACGACTGACCCATTAACAATGACTCGATGGATATCGTTTATAATTCCAACCTTTATGTCTAAATGT
GCTCCACACAGGTTA[CAA/C]CACACACACGGTCTGCTGTAGTGAATTTCATTGTGGAATGCTGATTGGCTAGAACTGTGTTCAT
GTGATCCGTAGTTAACCGCAAAACGATAACATCG

>LG11_12519192
CAAAACGATAACATCGTAGTTTAAGCCCAGCCTTGTCACAGTTCTAAATTAATGCTCTGAAACCAGGTCCTCTTTTCTCAGGCTA
CTACTCACATTGTTG[G/GTGT]TGTTAGCTATAGACTTGCAAAGACTATACTTTGAAATTCTACGATATAATTTAATGCGTGCCAC
AGTATGGATTATTGCTTGAAAAAAATTATGAATCCA

>LG11_12528838
CTATTATATGTCGAAGAACAGTGAAGTATACAACTTTGTGAGGCTACACACAGTGCATGTATGATAGATACAACTATGCATAAC
TAGATACAATGTGTGT[G/A]CACAATGTATCCAATTATCTATAACTACATACAGTGTGTGTATACGATATTAACTACATAGTAGT
TAAAATCGTATATGTAACTATGTATAACTATTTAC

>LG11_12528844
TATGTCGAAGAACAGTGAAGTATACAACTTTGTGAGGCTACACACAGTGCATGTATGATAGATACAACTATGCATAACTAGAT
ACAATGTGTGTGCACAA[T/C]GTATCCAATTATCTATAACTACATACAGTGTGTGTATACGATATTAACTACATAGTAGTTAAAA
TCGTATATGTAACTATGTATAACTATTTACCAGAAG

>LG11_12528845
ATGTCGAAGAACAGTGAAGTATACAACTTTGTGAGGCTACACACAGTGCATGTATGATAGATACAACTATGCATAACTAGATA
CAATGTGTGTGCACAAT[G/A]TATCCAATTATCTATAACTACATACAGTGTGTGTATACGATATTAACTACATAGTAGTTAAAAT
CGTATATGTAACTATGTATAACTATTTACCAGAAGT

>LG11_12528849
CGAAGAACAGTGAAGTATACAACTTTGTGAGGCTACACACAGTGCATGTATGATAGATACAACTATGCATAACTAGATACAAT
GTGTGTGCACAATGTAT[C/A]CAATTATCTATAACTACATACAGTGTGTGTATACGATATTAACTACATAGTAGTTAAAATCGTA
TATGTAACTATGTATAACTATTTACCAGAAGTGATA

>LG11_12528982
ACGATATTAACTACATAGTAGTTAAAATCGTATATGTAACTATGTATAACTATTTACCAGAAGTGATATATACAAGTCTGTATA
ACTACATACAGAATGC[T/G]TATGTGATGAATACAATTAAATATAATTTCATTCAGTGCATACTTATGCTCATGCATGTTTTAAT
GCATTAGTATGATTTCCATAGCGCGTCTTGTTCCA

Supplementary file 2: The X- and Y-sequences in gadMor2.1 assembly. X-sequence (pink) and Y-sequence (blue) are shown. The *zkY* gene sequence within the Y-sequence is highlighted in yellow, and the complex repeat sequence is underlined.

> X-sequence-and-flanks
ACACACACACACACACACACACACACACACACACACACACACACACACACACACACACACCACCCNNNNNNNNNNNNNNNNNNNNNCATTTATCTGA
CAAACAGTGGCTGACGGGGGTACTCTACATTTGTATTTTTTGCATTCTTTTTTTTTTCTTTTAAATGAGCTTTAAAAAAAAAATAAGATTCCACACA
CAAACACACCACATCCGCGCATATGCAGTGAGGGCGAGATCCTGCCTTGTGCTCTGTCGTTTCTCCATCTCTCTCCTCCACGTAGACCTGCAGCC
GCATGGTGGGGTTCTATCCTGTGGGACCTCGACGCCGCGGCGCTGCAAGCCACGTTCTTACCTCGCTAATGAAAATATCACAGGAGATGCATGG
AGACCCGGTGGGTGGTGATGACCTTTCCACAGAGCGAGAAAGAGGGAGGCATTTCGCCTCGCTGCTCTGCTCTCCCACTCCCTCACTCGCTCTCT
TGCTCTGTTGCTCTCTCTCGCTCTCTCTCTCTCTCTCTCTCTAAAAATCCCTCCCCCTTCACTCCCTCCCCCCTGGGGCGTTGTGGCAGAGCGACTG
GAACGTATTGAGTGCTTGTAGATCTATACTGTGTGTAAATAATTTATCACTGATGAGACCAGGGGAAAGTGCACAAAAATGCACCACAAAGGAC
GATGATCATGGTGGTGTGTGTGTGTGTGTGTGTGTGTGTGTGTGTGTGTGTGTGTGTGTGTGTGTGTGTGTGTGTGTGTGTGTGTGTGTGTGTGTG
TGTGTGTGTGTGTGTGCGTGTGTGTGTGTGTGTGTTGGTGAGAGAAAGCAAGATTTTAGCTATAAATTTAAATTTGAAAGGTTACATCTTAAATG
TTGTGCATCGGAGCCATGCCATTGTGTGGAAGCCTAAACCTTTTGAAAAAAAATCAACGCACAACGTGTGCCACGACTGACCCATTAACAATGA
CTCGATGGATATCGTTTATAATTCCAACCTTTATGTCTAAATGTGCTCCACACAGGTTACAACACACACACGGTCTGCTGTAGTGAATTTCATTGT
GGAATGCTGATTGGCTAGAACTGTGTTCATGTGATCCGTAGTTAACCGCAAAACGATAACATCGTAGTTTAAGCCCAGCCTTGACACAGATCTA
AATTAATGCTCTGAAACCAGGTCCTCTTTTCTCAGGCTACTACTCACATTGTTGGTGTTGTTAGCTATAGACTTGCAAAGACTATACTTTGAAATT
CTATGATATTATTTAATGCGTGCCACAGTATGGATTATTGCTTGAAAAAAATTATGAATGCTACGAATCCAACAGTAAAGTCCATATATGTCCCT
GTGCAGATATGCCCTTATAGTTGTTCGGGACAGTGTAATCAATGTGGCAATCGGCACATATTTCAAGACTTTGGCATAACTTAACGTGTTCATTC
TACCCATCTATTTTAATTATTCGCGGGCTGAGCTAACCTTTTACATAAGCCATCCCACTGTGCTTTGAAGAGCGGTATTCAGAGTAACATATAAC
ATGCAAACTCTGATTATTAATAATTTCTGCTGCCCAATGATTCATAATTATTCATTTGGAAAGTTCAAAATATTATAATAGGAAAAGAAAATTGC
TCTTATTAAAGAGGTGATTTATAAATTGTGTATTATATTGGATCACAATGTCAATGCATTTTGTACTCGCAGCACTAATAACTATGAAACTTTTGT
GCAAACATTGATGAGGTCACATTGGTTGCTTTTAGAGGCACTAACAACCGCATTGGCACACATTATTTATTTGTTTACACAGTGTTGTTTATTCAA
AGGTTCGTATGTCTCCACCATTCCTGCTGACTTATAGGATTGATGCAGCTGATCAATGTGAGTCAATGCGTACCAGTGCCCCATTAGCTTGGCAT
CCATTTGGCACACTTTTAGATACCCAGCGGAAGAAAATATGTCATTTGAAATTTAGCGTTTTGCATATTGCTAGATCATTGAATTTCGTGTTAAAT
TCTTTCCCATACACCCTATTATATGTCGAAGAACAGTGAAGTATACAACTTTGTGAGGCTACACACAGTGCATGTATGATAGATACAACTATGCA
TAACTAGATACAATGTGTGTGCACAATGTATCCAATTATCTATAACTACATACAGTGTGTGTATACGATATTAACTACATAGTAGTTAAAATCGT
ATATGTAACTATGTATAACTATTTACCAGAAGTGATATATACAAGTCTGTATAACTACATACAGAATGCTTATGTGATGAATACAATTAAATATA
ATTTCATTCAGTGCATACTTATGCTCATGCATGTTTTAATGCATTAGTATGATTTCCATAGCGCGTCTTGTTCCATTGTCTGGCGTCCTGGTCCAGA
TTGGCCTGGAGAACCTATTATGTGATGCCTGGCTCTCCCTTGGTACTTATCCCCATTACTATGTAATGACATTCATGAGCTGGGCACGCCATACG
GTGGCACGTGCTGCACAAGGCTCCTCACTATGGATCTAGTGTAGGGGCCGCCATAGTGTCCTCGGGCCATTTGGCTGAGGCCTAATGACCAATCC
TGTGCCCCACCCAAGATCCCCCGCCGCCCCCCACCCCCAGGTCCCAGGAGTGGAGGGCTGCCGAGGCTTTGTAGGAGAGGCACTGAGGACGCCT
TTGAAGGAATGTTCCGGGGCGCGTGGATGTTTGTGTGGTCGCCCCCGTTGCGTTCGGAACTGTTGTGTTGTGTTGTGGGAGGTGTTGTTTTCAGGT
GGTGTGTTGTGTTGTGCTGTTCTTGGACTTGTGTGGTGTGTTGTGATGTGTTGCATTTTGCACATACTGCCCCCCCANNNNNNNNNNNNNNNNNN
NNTCACAGTAGGGTCCTCTATAATAGGCCCCCTGCCATCTGGAGACCCTGTCCAGGGGGGCTCAAACAAAAGAGCACACTTATTATGGTAGAAT
TATATATTTCTGTGTGTGCGTATATGTGTGTGTGTCTTTGTGTTTGCGTTTGTGTGGTGTGTGTGTGTGTGTGTGTGTGTGTGTGTGTGTGTGTG
TGTGTGTGTGTCTGTGGCTTGCATGTGTGTGTTTGCGCTTCCCTAGGGTGTGTGTGTGTGTATGTGTCTGTGTGTGTGTGTGCTATGGCAAGACAT
ATCACCAGATCTGCTCCACCTAAACCAATGTTTTCTCACCCCCTGCATCCACCCTGGGGCTATTAGCTTATGATGTTCCTTAGCAGGCTAATGGGC
GCACACACTCTGGAGCGGAACCGCGTCTGGAACATCAGAGCTGGACATTAATATTTGGCA

> Y-sequence-and-flanks
ACACACACACACACACACACACACACACACACACACACACACACACACACACACACACACCACCCNNNNNNNNNNNNNNNNNNNNNCATTTATCTGA
CAAACAGTGGCTGACGGGGGTACTCTACATTTGTATTTTTTGCATTCTTTTTTTTTTCTTTTAAATGAGCTTTAAAAAAAAAATAAGATTCCACACA
CAAACACACCACATCCGCGCATATGCAGTGAGGGCGAGATCCTGCCTTGTGCTCTGTCGTTTCTCCATCTCTCTCCTCCACGTAGACCTGCAGCC
GCATGGTGGGGTTCTATCCTGTGGGACCTCGACGCCGCGGCGCTGCAAGCCACGTTCTTACCTCGCTAATGAAAATATCACAGGAGATGCATGG
AGACCCGGTGGGTGGTGATGACCTTTCCACAGAGCGAGAAAGAGGGAGGCATTTCGCCTCGCTGCTCTGCTCTCCCACTCCCTCACTCGCTCTCT
TGCTCTGTTGCTCTCTCTCGCTCTCTCTCTCTCTCTCTCTCTAAAAATCCCTCCCCCTTCACTCCCTCCCCCCTGGGGCGTTGTGGCAGAGCGACTG
GAACGTATTGAGTGCTTGTAGATCTATACTGTGTGTAAATAATTTATCACTGATGAGACCAGGGGAAAGTGCACAAAAATGCACCACAAAGGAC
GATGATCATGGTGGTGTGTGTGTGTGTGTGTGTGTGTGTGTGTGTGTGTGTGTGTGTGTGTGTGTGTGTGTGTGTGTGTGTGTGTGTGTGTGTGTG
TGTGTGTGTGTGTGTGCGTGTGTGTGTGTGTGTGTTGGTGAGAGAAAGCAAGATTTTAGCTATAAATTTAAATTTGAAAGGTTACATCTTAAATG
TTGTGCATCGGAGCCATGCCATTGTGTGGAAGCCTAAACCTTTTGAAAAAAAATCAACGCACAACGTGTGCCACGACTGACCCATTAACAATGA
CTCGATGGATATCGTTTATAATTCCAACCTTTATGTCTAAATGTGCTCCACACAGGTTACAACACACACACGGTCTGCTGTAGTGAATTTCATTGT
GGAATGCTGATTGGCTAGAACTGTGTTCATGTGATCCGTAGTTAACCGCAAAACGATAACATCGTAGTTTAAGCCCAGCCTTGTCACAGTTCTAA
ATTAATGCTCTGAAACCAGGTCCTCTTTTCTCAGGCTACTACTCACATTGTTGGTGTTAGCTATAGACTTGCAAAGACTATACTTTGAAATTCTAC
GATATAATTTAATGCGTGCCACAGTATGGATTATTGCTTGAAAAAAATTATGAATCCATGCTACGAATCCAACATTAAAGTCCATATATGTCCCT
GTGCAGATATGTAAGGGATAATGAATGTGTGAATTACGTTACAATTCCCTCCTTGATCATCTCAGATGACCATCCTATAAGCAAATAGAAAAAT
GCATAAAACGTATAAATGTATAAAGGTATAAAAGTAGAAAGGTACTAAAGTATAAAATGTATAAATGTATAAATGTAAAAAGGTACAAAAGTA
TAAAAGTATAAAAAGGTACAAAAGTATCAAAGTGTAAAAGTATAAAATCATCAACACCACCAATCATTATGGGAAAACACACCTTATTGGAGT
GAAAACCACAACCCGTCCCCCTAATCCTGAAACGGAATTCACCTTGGTGGGTCTCACAGGAAATACAGACCAGTGTCAACAATACAACAGTAGG
ATCATATGGAATACACAGAAATTAATAATGACTAAATTAATAATCAAATAGTAAATCAATAATCAGTAGTAAAGCAATAATAGATCATTAACCA
AACAGTAAATCAATAATCAGTAGTAAAGCAATAATAAATCATTAACCATAGAGTAAATCAATAATAAATCAATAACAAAACAATAAATCAATA
ATCGGTAGTAAAGAAATAATAAATCATTAACCAAACAGTAAATCAATAATAAATCATTAACCAAACAGTAAATCAATAATCAGTAGTAAAGCA
ATAATAAATCATTAACCAAACAGTCAATCAATAATCAGTATTAAGCAATAATAAATCATTAACCATAGATCCAAAAGGGCACACATATAGGTGT
AATCATAAAAAATAAGAAACATAAACACAACAAATAACTCTAAATATATGTGACGCAAAGTCCTACACTTTCAGCATGCATTGAAAGTCAACAC
CACAAAAAATTATTAAATAACCACAGCATGCTAATGGTCACAATCTATCCTCTCCAACCATGGCCTCCCAGCACAGATATCTACACCACCGTTCG
CTTTTATGGAAGGTAGCACAAAATCACTTAACATCGCCTGGAAAAAATGAAAGGTTCTACCACACTATCAATCCCAGCCTAAACCACAGTCCTG
GGCTGGTATCCATACTGAATGATTTCGATTCAGGCTTCATCTTTCACCAGGCCAGAGACAATCTTTTGGGCCTGGTTCAGACGTGATTGTGTGTGT
GTAAGGGCGTTTCAGTGTTGGGGTGACGGCCAAGGGGTTTGGGTTGGAAATGACCTCTGTAGTTTGTGGTGGGGTCCGCCTCCATATATTATTTA
TCCAATCAACATTTTTGTTAATAGTGGACCACCAAAACAGGACTGATTCGAACCCTGCCGAAATTTGATTGCATCATCCTTGGGTAGTTGGTCGG
CTCCGAGCGTGTCTGCATCTGGCTCCTCCTCCGGCCCTGGAACTTTGTGTTTCTCTTGCTGGGACTACAAACTTATGTACAACAGTTTAGATGTTC
AAACATATTTACCTTAACTCTATTTCTAATTACTCTAATGGAAGTCCCTTTTGGGACCTTCGAATGTTAAGTGAAGGCATGGGTCGACCCTTAAGC
ATTTCATGCGGGGTTAGATGCGTCGTTCTGTTAGTTTTCATGTGATAACTCATTAGTGTCAGCCATAGTGCATTAGTCCTTTAATTTAGTACTCTA
ATTTAAATTTGTCCTTGACCAAAGAGATATACCCCATATTTTAATAGGTGTAGTCCTACTTGTACAGTTTATCAAGATTACGTTTTTTTTTTTTTAA

TTATTATTCTCATCTTTATCATATCCATATTTATTCAATTATTCTTTCCTGTGTGAAAATAATAATAATCACTTAGTTAATAGCAACACCTTCTTTA
GTCCAATTGCATTAGATTATCATCACTAAGCGTCTTAGAGTACCATATTAAGCGCTATATAAATTTCATTTATTATTATTATTATCACTGTTGTCCT
ATTAAAGTATCAACGTTATTGTCCTTATAACCTCCTCATGAACTTCGATGTTCATCATCATGAATGATAACTGTTGTCCTAGGAAGACAAAAAAC
AAAACTAAAACTAAAACAAATTAGCCATAATGTGAATATCCACTGCGTGTCTCCCTGTGACTCTCTCCCTCCTTCAGGAGAACACGCTCTATCTC
CCTCCTCAGTACGGGGTGCTGTTTGATGCAAGACAACCGATAAGGTTGGAATGTGGCTTCTCTCTCTCTCTGCGTGTCTCCCTCCCACGTTGGGTG
CGGCCATAACGCCTTGAAGCGTGCGTGCTCCGGGGGCTGGGTGTGGGAGGAACCAGTGGTAGTTCCTTTTCTCGGCCAACAGAGGGGTTGTTCA
GGTGAAACCTGTGGACAGAGACTACAAAGAACTTCAAACATGGCAGACAGGGAACTACAAATTTATCAAAGCCTGAAAACTCAACAGAGCTCT
ACCTGCATTCCTTGGATATGTGGTATTTTATGGCATATGGCCACCACACTCTTTCAGTTGAACACACTCTCTTTTTCCTTTCTCCGTACTGTGTGTT
AGTGTGTCTCTCTGTACTGCCTGAATGGTGTAGGTCGACAAATGGGGGGGAAATGTGTTCTCTCTCTCTCACCCTTCCTCACTCCCCTTAGGGTCA
AGCAGCACCTAGGACCGTTAACAGATAAGCCTGGAATGCAACATCCTCAACTCCCCATCATGGAACAAGGCCTAATAACTATCTCTCACACACA
AAAAGATCCTTCTGTCCCTTCATTCACAATCACATCTTGCTGCATCCATTCCCAAATAACTTTTCACTTTATTATAAAGAACTTTACTGTTGCTCCC
CCTTTCCAAATTTCTATATGCACAGTTTTCAAAACTACAAAGCTGACCACCGAAATTGGTACAGGCCTCGTTAGGGCAGGCCGAACCAAAATAC
CCTCACAGGTTCTAACTTCCATACAATTCTAGGGAGGGGAAGTACAACATGAGGTGAAGTAGAGGACTTCAAATATGGGGAACGGGGACTAAA
AGATCTCGGGGCCTGACAGCTAAACAGAGTCTCTGTCCGTGTTCCTGAAGGAACCGTCCACTCTCAGAAGAACACCCTGTCTATCGCTACCTCTA
ACAGACTAGAGCAGGGAAAATGGTCCCTCTCAAATGTCCTCCGACTTTAGCTCTCCTTGTCCATCCCTCTCGCTCCCTCTCACACAAATGCACAT
TGACATGTACATGCTGTCACCCACATTCACACTGACTTCACACACGGCTTCTCCCGTACCCACAAAGATTAACAAACATCAATATGAACAGATCA
TAGAGTCAGACAACCACACTCCATTAGCAGCCAGGAGGCTTGCTCCTCTATCTGCTACAGTGCACCTCTCACACACACACACACACACACACAC
AGAAACATCACAACAGACCTCCCTCATTCTGTCTCCCGCAGACGCAGTTTAGAAATAAGCTGAAATCTAACATTTGGTGCCGTGACACCGTCTTG
AAGAGAGAGGAGTGAGAAGACGATTGCCTCTGAAGATTGCCCTGTGATCGAGAAGACCGCGCGCCCTGCCTGGGAAGACGTCTGATCAACGCC
TGCCCTGCTGAGGGACCCGCATCCACAACACGGCTGAACCAGCGCATCTCCATGCACCACGTGGGAATCAACGTTGACTGAATCTCATTCCACA
CTCTCGACCAAGAGAAAAGAACAAGAAAGTAATCTTAAAATAATCTACACACACATAACTGTTATTATTATTATTTGTTTTACTATTAGGCTGCA
CTACATTTGCTGAGTAATGAAGTTGTGTCATTAAGAGCTTGACGAAGTGAGAACCTATATTAGTGATTAAGAGCTCAATAATCACTGAATATACG
CCATATGAAACTCTTTTGAATTTGTTTGTTGAATAAGGCCTTTGGTCTGGGCTAGAGTCCTAGAAACTGGGCTAGAGTCCTAGAAGCTGGGTTAG
AGTCCTAGAATATTGTAATAGCATGTCATCCAAGTGATTTGTTCATCTTTGCTAGTTTGTCAATGTTGATACGTGTCCTGAATATAACTTAAGACG
TTTGGTTTGCTCAGGGGTTTTTGTTTTACGACTTGTCAGACAAGGAGTGAAACCGTATTTGTATTGAATTCACTGGTGAGGTTACATGAATCGCTA
GAGTTTTTTTTTGTTTGTGCTATGTTGTTTTGATCCCCGCCGAGGAATCACATATATTGGTTTTTTTTAATCGAAGAAGGAAAAAGTGTGCCATCA
TCTGTTGTTGGCATACCGAGCTCTGTGGGAGGCGTCTATTTGTTTCTTTCTAAGCAGCGATTGGTCGCTAGTTTGAGTTACTTTACAGATAGAAAA
AGATAGAAAAATGCCGAAAGGTAAGGGCAGGAAGAAGGCAGAGGAGGAGGAAGAGGATAGCAATCTGAACCAATGGAACAAGATTCTTTTGA
AGTGGAGGTTAGAGAGATTGATCTTTATTCAGTTAGCGAGACTCTGTAACAAAAAGAGAGATGAATTGGAAAAGAACTCGGAACAAAGAGCAG
AGGAAGTTCACAGACCTACACACGACGCTAGTTAGAGAAGGACTGATAGCGGCCCGCCCCCTCGACTCAGGGGGGGGTGACCAAGGCAACGGA
GGAGGAACCTTGGGAGTCTATGGTCGCATGTACCCCACACTGCCATCGTGTGGCCCAGAAGGGGAATACATCTTCCCGATGCAGCCATCGACGC
CATCTGGTGGTTTACAGGAGTCAAGACACAGTGTTCTCTCAGACGTCAACAGCTTTATAGGCGAGTACCCGGTCATCTGCAGTACCCCCCACGTG
CCACGGAAGGGAAATCCATCTGCCCCACCTGACTATGCTAATCATGATGCGATCCAGATCTCACAACAATTTCAACATCCGAACCCCCCAACAC
AACAACCCCCCGAGGTGAGGCCTGGAACAGTGACTAAGAATGAATCACGCATTGGAGGATTTGATGACAACAAACAACCACACTGCAGCGCAA
CGGGAGGCGCAGCCACACCACACATGACGACCAAAGATGAGAAAGACGAAATATTTAACCTCAGGCCCTTGAAGAAGATAAAAAGCGCTGAG
GGACGCACAATCCTCTATGAACCCTCCAGCCCGAAGGACATCGAAAGATGGAGTGCGGAAATCGAACACCCGAGGAGAACAGGCCTACAGCCA
TGGATGAAATTGAAACAACTGATGCTTCTGTACGAACTCCACCCCTACGATGGCCTTGCTATATTGTTCAAACACCTGAGCAGCAATGAGCGCAC
CCAGATTCGATACGAGGTGGAAGATGCCATTGGAGAAGACGGAATGAGACCAGGAAAGGGATGGGAGGCCATAAACGCGTGGATCCAAAAAC
ACTCCAGGGCACAAGTCAACTGGACAACAATCACTCGTTGTATGCAGAAGGAAGACGAGGGTCTGGACCAGTTCAATAAGCGCTTCTTCGAATG
TTATTTGCTGTACTCAGGCCAAACTGAATATGATATGGACACCATCGACCAGTCACAGGACTTGCCTTTGAAGACCATGTATCTCCAGCAGGTAC
TGCCGGAGATCCGAAGGGGAATAAAGACAAGGTTTCCCGGCTGGGACAACCAAAGCAGCACAATGGCAGAGATCAAGGAATTAGGAGAAAAG
GTGGATCGAGATGAAGAAGTCAGGATTCGATTCCTGGCTGACGAGAAGAAGGGAATGGCCAGGGAACGAGGAAGATTCCCCCAGTCCAACAGG
GACAGAGGACGATTCCCCCAGTCAGACAGACAGAAAGAACCTCGACCATGCCACAACTGTGGACAAATTGGACACTGGATGAGAGAATGTAAA
GCCCCTCGAAAGATGTACCGTGACCGCTCCAACGAGAGAGTAGGGGAAGAAAACCGCTCAGACAAAATGAGGGAACGATTGAAGAAGTTACA
GGCGAAGAGTGTTGAAGAACTCAGCCGCCTGTGCGATTCAGTGTTGGAAACCAACAATAACACCAACTTGGAATGACACTCGTTAGATTTGGCA
CGCAAACTAACTGATACGGGGTGAAGAGTAGGGAGTGTTTGAGTGTTTGTTATTTTCATTCCTTTATTTTCACAAGAGGTTAGTCTTCGAAAGAT
GCTAGGACTTTTGTCACTGCCTATCTAAGTTCATGTTGGTTCCTTCTTGACACAACTGCTCAGCTTTAGAACATTTTGTTTGTTTTGTTTTATTTTTG
AACAAAGGTTCAATCATTCATTCATTCACACAAGGTTTATGGAGAACTATGATGACTGATTACTGATTACTGATATAACCATTTTTGAATCAAGC
GATTTATTTTTGTATGATCATTTTTTTCATTGTTTATCGAATCATTAAGACAATTACAAACCATAGTTGATTATTATTACTGTTGGAGGCTGTGACA
ATGCCTCCCCCATGACTGATACTGCACTTTTTCTTTGTTTCATGTTTCATCGTATCTGGGAGATCCAGGAGACACGGACACTTGATGAGTCCTTGA
ACTGAACTGTGTCTGTGTTTGTCCTGACGCCCTACTACGAACTTTCATCCTCATCCCCCATCACACCTGTGGTGGCCATCAACTGGAAAAGAACT
CTGTTGCAATTGCTACTCAAAACCAACTGAGTCATGCAGTATTGTCTGTGATGTTGTTGTGCCAAGCAGAGGGAGGTGTCTGGACAGTTGACTTT
CTTGATTCTTTAAACTTTTTATGTTTTGGTTTAATCATGAGCAACCTAATGAGTTTGTTATTTTCTTTATTATAGTGGCCTACACCTGGTTTACTTGT
GTATGATTTGAATTTACTTGTCCTATTGCCTTTTATGCTTTCTTTCATTGATTTTGTTAATTATTAATGGGTCACCAGTAAATACACTGAATTCTCA
TTTTTTGGGTGATCTTTAAACTATGTTTAGTATGATCAAGAGGGAGGAATGTAGAATGTTGGTGTATTTTAGTGTCTCTCTTTTGTTCACATTTCTA
GTCTAGGAACAGGCAAGGGCCTCTCATACTGATACAAGGTGTTATCCCAAACATTCTGCCATTGTTATGTTTCAACGAGGTTGAACCGACCTGGT
CCTCTGGGACATAGCTAGGGCCAGATACCTTATCAAGGCTGAGAGTGCGTCTGTTCACGTGTTATTCATGTATCCCCTTTTTGTATAATACTCGCC
CCAACCCTTTGGTTCGAGGAGTAGAGGGTTTCGACAGCCAAGGAACAAGTCATCGACCACCGGGTCCACTATAGATTATTCAACCTAAGTCTGT
ATCTCGCTGTATTCCTGGAAAATAATCTATTTAACCCTCTAATCCAACCTGCCAAGCTGCTTTGATTTCGACTTCAAGAATTACTACTACGACTGA
AAATACACAACGCAGAGTCTGTCCGAACAGTTTAGAAATAAGCTGAAATCTAACAGCACACACATATAAACATCACCTCTTGCATACCCACGCA
CGCACAGAATCTCTCAGACTTTCTCCTCCAAGCGCACACACATAAACATCGCCTCTTGCGTACCCACACACAGAATCTCTCAGACTTTTTCCTCC
AAGCGCACACACATAAACATCGCCTCTTGCGTACCCACGCACAGAATCTCTCAGCATCCATAGCAACATAGCGTTAAGCATTTACATAGCAAAA
ATGTGTCAACACATAGCAGGGTTAGTCACTCAAAAAGTAATTACATTGGTCCAAACCATATAATTCGAACTGGCCGCAATCGGCCCCGAGACAT
ACAAGTCCGAACATTATAGTCGAACTGGCCGCTATCGGCCCGAGACATACAAGTCCGAACAATATAGTGAACTGGCCGCAATCAGCCCCGAGA
CATACAAGTCCGAACATTATAGTCGAACTGGCCGCTATCGGCCCCGAGACATACAAGTCCGAACATTATAGTCGAACTGGCCGCTATCGGCCCC
GAGACATACAAGTCCGAACATTATAGTCGAACTGGCCGCTATCGGCCCTGAGACTTACAAGTCCGAACATTATAGTCGAACTGGCCGCAATCGG
CCCCGAGACATACAAGTCCGAACATTATAGTCGAACTGGCCGCTATCGGCCCCGAGACATACCAGTCCGAACATTATGGTCGAACTGGCCGCAA
TCGGCCCCGAGACATACAAGTCCGAACATTATAATCGAACTATAACATTAATTTATGTATACAATTCTGGCCCTTTGCTTCCTTCAGCTAGTCTCG
CAATGGCGACAAAGAACATTATTTTAATTCAATCTCCTTCAGAGTCCTCAGCGACTGTTCAACTTCACGTTGATTACACTTTTTCTACCAAGCCGG
GATTCCAGAGCGTACACGGCAGGTCGTTGGCTTCCACCACTTCCACCTAAAATTTCTAAATCTAATCCTATGCAGATTTAGTCTTTAAGACAATA
GGTTCTGCTTACCTGTTTTGTGGCCACTGTGGAAGGGATGGTGAGCCACGCCCGTCGCTCGCCTCTGCAGCTCCCCCGGTCCTTGGCCTTTCCTCA
GAATCACGTCGGGGTCACCAAATTGCCGTGTTCTTTTGGAAAAAGTCTGCCAAGTCAGGATCGCTTTAACTCACTTTTTAGCAAAGGTTTGGACT
ACCATACTATGAAGCAAAAGGAGGGGAATTGTACAAACACGTGTGGAGATACACTTAGTAGGGCATTCAAAAGGATGCTTTACCTGGAGCGTTC
TAACCCCCTGGGCTATGTGTTGGGACTTATCTACTGGGCAGATGTGGACTCAGTTATGTGCTCTGATTGGCTAAGCATGGTGTCTCTATGCTGCTC
CCTTGTGGCTATGTGTTGGCATTGCAATTTGTTATGTGGCCCTGAATTTCTGAGTTCATCGAACACCTGGGCCGCACGTATCTGTAGAATTGACAC
ATGACGAATGGCCTCCTTTATGAGCTGGACGCCTCCCTAGGCTCCGGATCCCTCCTTAGCGTGATAAAGAAGACTCTTTCATTGTTAAGGGCATA

TGTGGCTTTGGTATGAGTGTGTGAATTACGTTACATGGCTATGTGCGGGAATTACACTTGTTCTTGTGGCCTTGAGCGTCTGGGTCTCTAAAACAT
CCTGACCGCTCGTATCTCTAGAATTGACACATGATGGATGGCCTCCTGTATGAGCTGGACGCCTCCCTAGGCTCCGGATCTCTCCTTAGCGTGAT
AAAGAAGACTCTTTAATTGGTAAAGGCATGTGTGGCCTTGGTATGAATGTGTGAATTACGTTACAGCACACATTGTTTATTTATTTACACAGTGT
TGTTTATTCAAAGGTTCGTATGTCTCCACCATTCCTGCTGACTTATAGGATTGATTGCAGCTGATCAATGTGAGTCAATGCGTACCAGTGCCCCAT
TAGCTTGGCATCCATTTGGCACACTTTTAGATACCCAGCGGAAGAAAATATGTCATTTGAAATTTAGCGTTTTGCATATTGCTAGATCATTGAATT
TCGTGTTAAATTCTTTCCCATACACCCTATTATATGTCGAAGAACAGTGAAGTATACAACTTTGTGAGGCTACACACAGTGCATGTATGATAGAT
ACAACTATGCATAACTAGATACAATGTGTGTGCACAATGTATCCAATTATCTATAACTACATACAGTGTGTGTATACGATATTAACTACATAGTA
GTTAAAATCGTATATGTAACTATGTATAACTATTTACCAGAAGTGATATATACAAGTCTGTATAACTACATACAGAATGCTTATGTGATGAATAC
AATTAAATATAATTTCATTCAGTGCATACTTATGCTCATGCATGTTTTAATGCATTAGTATGATTTCCATAGCGCGTCTTGTTCCATTGTCTGGCGT
CCTGGTCCAGATTGGCCTGGAGAACCTATTATGTGATGCCTGGCTCTCCCTTGGTACTTATCCCCATTACTATGTAATGACATTCATGAGCTGGGC
ACGCCATACGGTGGCACGTGCTGCACAAGGCTCCTCACTATGGATCTAGTGTAGGGGCCGCCATAGTGTCCTCGGGCCATTTGGCTGAGGCCTA
ATGACCAATCCTGTGCCCCACCCAAGATCCCCCGCCGCCCCCCACCCCCAGGTCCCAGGAGTGGAGGGCTGCCGAGGCTTTGTAGGAGAGGCAC
TGAGGACGCCTTTGAAGGAATGTTCCGGGGCGCGTGGATGTTTGTGTGGTCGCCCCCGTTGCGTTCGGAACTGTTGTGTTGTGTTGTGGGAGGTG
TTGTTTTCAGGTGGTGTGTTGTGTTGTGCTGTTCTTGGACTTGTGTGGTGTGTTGTGATGTGTTGCATTTTGCACATACTGCCCCCCCANNNNNNN
NNNNNNNNNNNNNTCACAGTAGGGTCCTCTATAATAGGCCCCCTGCCATCTGGAGACCCTGTCCAGGGGGGCTCAAACAAAAGAGCACACTTA
TTATGG

Supplementary file 3: Oxford nanopore sequences of Atlantic cod that aligned to the X- and Y- sequences in the gadMor2.1 assembly in fasta format.

>c306e846-98b4-4aec-8e8e-b3bc32d8f90b runid=70d2f0943bc097aba7d2e385cddaea56c3683cfe read=2082 ch=275 start_time=2017-05-11T10:19:07Z
GTGTGCATTGGTTACGTATTACTGTTTCGCATTATCATTGAAGCAACAGAATTTTCATTACTTCAGAAGAACTTTTGCATCTCGACTTCTACAGAA
CTAGGTGCAATTCATGAATGTTGGCGTCGTGGATCAGTAGATCGCCATTCCCTCATTTGTCTCCGTTGGTACTCACAGCCTCCCATTAATCACCAG
TCTGTCTTCCTCCTCCTCTACCCGCTCTTTATTCACATTCCTTCTTGGCGTAGATCACGGCTGGTGGCCTCAGATTCTGATTTTTCACTAAAAAGCT
CGAGGGAAGGGGCAGAGAGGAAGGAGGGAGAAGCAGGAGAGGAGGGAGGAAGAAGGAGGAGGAGGGAGGGGTGAGTTGTGAGTATTTTCAT
ATGAGACAAGGGACAAGGACAGTGGTTCTTAATTCCGTGTTTTGGGAAAAAATCTGCCATCCTCCTTTGATTCTGTTTTGGGCCGCGCTTTGAAA
AGAGAAGGCGGGGGCGGAACAATAGTGCCGTTACTTCACCGGGGAGGGGTATTCCAAGTGTGGAGGAAAAAAATCCACATTTATGTAGCAGAG
CAATATAACGTTAAGTCTATGAGGGTGGTGGTTTTCCAAGTATATATCTGAGAAAAAGCGGAAAATCACAAACAAAGTCGGGGCAAAAGCAAC
TGCCACTTGGTCCCTGACACTTTAGATCACTGCATACGTCCTAAATAATATGGATTAACATCACAACACAGCATCAAATCTGTTCTGGATGACAA
AGTCAACGGCTCGAAGATGTTGCAGTGAATATAAGATATTGCAATAATGCAATGGTAGTTGTACTTGCTATTGCCTTTCTACTGTTGATAGTGAT
GGAATATACTGACTAACCTATACTATGCATGAATACCATAGTAACTTGGTTACCCTGTCTATTCCTAGACAGAAACTGTACCAAATATTAATCAG
CTCTGATGTTCCGAACCTTCGGTTCCGCTCCAAAATTATTGGCCATTAGCCTGCTAGAACATCATATTGCTCTTAGGGATGCGGGGTGAGAAAAC
GTGGTTTAGGTGGGCAGATCTGGTGATATGTCTTGCCATAGCACACACACACACAGACACACTACACACACCCTAGGGGCATAAAACACACATG
CAAGCCACAGACACACACACACACACACACACACACACACACCACACAAGCATGAACACAAAGACACACACACATATCTTGCTTTACAAATAT
AATTCTACCATAATAAGTGTGCTCTTTGTTTTGAACCCCCTGGACAAGATTCTCCCAGATGGCAGGGGCCTATGAAGAGGGACCACAGACTGGA
TTTGTGTGTGTGTGAAATGATAATGTCAATAATGTAATGATATTGTGTATGTGTTGTATGCTAGTGTATACATATCTGTTATATGTGTTAGGCTAA
TCTTCTGGGTGTGTCCCTGTCTGCAGGGAAGGGGGGGGGGGGGGGGTGTAGCACGCGCGGGGGGGGTGGGGGGGCAGTGTGTGCAGAGCCAA
CACATCACAACACCACACAAGATCCAAGAACAGCTAACTGTACCACCTGAAAACAACACCTCCCACACAACACAACCTGGTTCACGAGCACCA
GCAGGCGACCACACCACAAACATCCACAGCCCCGGAACATTTCCTTCAAGGCGTCCTCGGTGCCTCTCCCAAAAGCCTCGGCAGCCCTCCCACT
CCTGGGACCTGGGGCCAGGGGTGGCGAGATCTGGGGCACAAGTTGAGATCATGCATTTTCAGCCAATGGCCCGAGGATTTTATGGCGGCCCCTA
CATAGATCCCTGTGTGCGCTTTTGACATGGCGTGCCCAATAACAGATGTCATTATAGTAATGGGGATAAGTACCAGGAGAGCCAGGCATCACAT
GGTTCTCCAGGCTTAATCTGGACCAGGGCGTAGACAATGAACAAGAGCAGCTATGATCATACTAATGCACATTAAAACATGCATGGTATATATG
CACTGAATGAGAAATTATATTTAATATATTCATCACATACATTCTGTATGTAAATTGTTTTGAACTTGTATATCAGGTAAATAGTTACATAGTTAC
ATGTACGGGTGCAACTTTACTATTGATTGGCCAACTTCGTCTCCCCTTTACTAATACAATATTGGTTATGAATGATTGTATGTGTTGTGTATCTGT
GAATTATACAGTTTATCTATCCTTTACATGCACTGTGTACTCACAAAGTTGTATACTTCACTGTTCTTCTAGGCGACATATAATAAGTTGTAGGAA
AATTTAACACGAAATTCAGCGATTTAGCAATATGCAGCTAATTTCAAATGACATTTTTTTCTTCCCGCTGGTATCAAAAATTATTGCTTAAATGGA
TGCCAAGCTAATAGGGCACTGGTGGCTGTGTCCACATTGATGGCTACGGTAATCCTATAAAGTCAACTTGGAATGGTGGAACCTTACAGAAACT
GAGACTCATTAAGCTGTGTAAACAAATAAATAATGTGTGCCAATACAGTTGTTGAGTGCCTCTAAAAAACAACCAATGTGACCTCATCAATGTTT
ACAAAAAGTTTCATAGATTATTAGTGCTGCAGTACAAATGCATTATTGTAGGTCAATACTAATACAATTTATAAATCACCTCTTAATAAGAGCAA
TTTCTTTTTCCTATTATAACCTTTTGAACTTTCCAATGAATAATTATGAATCATTGGGCATGAAATTAATAATAATCCCAGAGTTTGCATGTTATA
ACACCTGAATACCCATCTCTTCACAAAAGCGGCATTGGGATTAAACATGTAAGGTTGAAAACTCAGCCAGCGAATAATTAAAATAGATGGAACA
GAATGAACCGTTGGTTATGCAAAGTCTTGAAATATGTGCGATTGCTGATTACTGTCCCCGGTTTCTTAT

>e16f9ced-f786-4ee9-9205-de928135a1ff runid=7f584f21e80684e7350ead5ba688aa70fcd47957 read=40575 ch=347 start_time=2017-08-11T06:59:04Z
TCGTACTACGTTCCGGTGCCATTATTGCTCGCGAATCTCACAGCAGTACCTGCTGGGAGATACATGATCTTCAAAAGGCAAGTCCTGTATTTGGT
CGATGGTGTCTATCATATTCAGTTTAGCTTCAGGCACAGCAAATAACATTCGAAGCGCAACGAACTGGTCAGGCCACCAGTCTTCCGCTTCTGCG
CGCCTAGCAGTGATTGTTGTCCAGTTGACTGTGCCCTAAGGTGTTTTTGAGATCCCGGCATTTGCTGGCCTCCCATCCCCTTTCTGGTCTCATTCG
TCTTCTCCATGGCATCTCTTCCACCTGTTATCGAATCTGGAGTGCATCATTGCTGCTCGGGTATTTGGTAAGGCCATCGCCTTGATTCGTACAAAC
ATCAGTTGTTGCTCAATTTCATCATGGCTGTGGCCTGTTCTCCTCGGGTGTTCGTTTCCAGCACTCCATCTTTGATGTCATAACCGGGCTGGAGGG
TTCATAGGATTGTCGTCCTCAGCCTTTTGTCTTCTTCAAGGGCCTGAGGTTAAATATTCTAATCTCATCTTTGTTAGATCATGTGAATTGTGGCTGT
CGCCTCGTTCGCGCTACCATGAGTGTGGTTGTTTGTTGTCATCAAATCCTCCAATAAGTATTGATTCATTCTTAGTCCTGTTCAGGCCTCACCTTTC
GGGGAGGTTGTGTGTGTTTTGATTCCGGATGTTGAAATTGTTGTGAAGTCTGGATGCATCATGATTAGCATAGTCAGGTGGGCAGATGGATTTTC
CCTTCCGTCAACGCGTGGAGTACTGCAGATGACGGTGCTAGCCTATAAAAGCTGTTGACGTCTGAGAACACTGTGACATTGACTCCTGTAAACC
ACAGATATCGACCTTGGCTGCATCAGGGAAGATATATTATTCTGGGCCACACGATAGTGTGGGGTACATGCGACTCAGAGCTCTGGTTTCTCTCC
TCCGTTGCGCTTGGTCACCCAGGGCCCTGAATCTGAGGGGCCGGGCAGCTATCAGTCCGCCTCTAACTTCAGGTGATGTGGGTCTGTGAACTTCC
TCTATCTTTGTTCCGGTTCTTTCAATTCATCTCTCTTTTTGTTGCAGAGTCTAAGCTAACTGAATAAAGATCAATCTCTCTAACCTCCATAAAAGA
ATCTTGTTCATTAGTTCCCTTTCATCCTCTTCCTCCTCTCCTCTATAACTGCCGCGCCTTACCTTTCAGCATTTTCTGCTCTTTTTTCTATCTGTAAA
GTAACTCAAACGCGACCAATCGCTGTAGAAAGAAACAAATAGGCAGCCTCCCACAGAGCTCGGTATGCCAACAACAGATGGCACTTTTCCTTCT
TCGATTAAAAAAAACCAATATATGATTCACCGTGATCAAAACAACATAGCACAAACAAAAAAAAACTCTGGCGATTCATGTAACCTCACCAGTGA
GATTCAATACAAATCACGGTTTCACTCTAACCAAGTCGTAAAACAAAACCCTAGAACAAATAAGCATCTTAAGTTATATTCAGGACGTATCAAC
ATTGACAAACTAAGCAAAGATGAACAAATCACTTGGATGGGTATGCTATTACAATATTCTGGGACTCTAAGCAGCTTCTGGGACTCTAAGCCCA
GTTCTAGGACTCTATTAGTAAAGGCCTTATTCAACAAACAAATTCAAGGGTTCATATATAGTATATCTTCAGTGTGGGCTCTTAATCACCTAATA
TGGGTTCTCGCTAGATCAAGCTCTTAATGACTACAAACTTCATTACTCAGCAAATGTCTCAGTGTACAGCCTATAGTAAAATAAGTAATAATAAT
ATAAGTTAATATTATTGTTAATGGATATTTTTAAGAATTACTTTCTTGTGCTTTCTATTGGTCGAGGGTGTGAATGAAGATTCAGTCAACGCGTTG
ATTCCCACGTGGTGCATGGGAATGCGCTGGTTCAGCCGTGTTGGCGGATGCGGGTCCTCAGCAGGGCAGGCGTTGATCGAGCACAGGCAGAGGC
GCGTTCTTCGATCTGGGAACGATATTCAGAAGCAATAAGTCTTCTCCTCCTCTCTCTCTCTTCAAAGGCGGTGTCCGCGGCGCAAATGTTAATTTC
TAGCTTATTTCTAACTTCGTCTGCGAGAACCTTAGAATAGGGGTTCTGTTGTGATGTTTCTGTGTGTGTGTGTGTATTGTTGTGAGAGGTGCACTG
CTGCCGATAGAAGAACCAGCCTCTGGCTGCTAATGGAGTGTGGTTATCTGACTCACCGATCTGTTCATATTGATGTTTTGTTATCTTTGAAAAGC
AGCAGGGGTCTGGGAGAAGCAAGTGTGTGTAGAGTCAGTGTGAGATGTGGGTGACAACATGTACACCTCAATCAATGTGGTTTGTAGAGAGGC
GAGAGGGATGGACAAGGAGAGCTAAAGTCAGGAGGACATTGAGAGGACCATTTTCCTGCTCCCTAGTCTGTTAGAATTAGCGACAGACAGGGT
GTTCTTCTGAGGCCGGTTCAACTAAGGAACTGGACAGAGACTCTGTTGCTTCAATCAGGCCCCGAGATCTTTGGATCCCCGTTCCCCCACTTTGA
GTCCTCTACTTCACCTCATGTTTGTGCCTTTCCCTCCCATAAATTGTATGGAATGAGAATTATTTTGGTTCGGCCTGCACTAACAGAGACCTCTGT
ATAGACAGTCAGCTTTGTAGTTTTTTAGAAAACTGTGCATATCTCAGAAATTTGGAAGGAACCTTAGTAAAGATTCTTTATAATAAAAGTGAAAA
GTTGTTGGGGAGAATGGATGTACAGCAAGATGTGATTTAATGAGAATGAAGGGACAGAAGGATCTTTTATTGTGTGAGAGATAGTTATTAGGCC
ACTTGGTGTTCCATGATGGGGAGTTGAGGATGTTGCATTCCGACTTATCATTAACGATTCCCTAGGTGCTGCTGACCCTAAGAGTGAGGAAGGAA
TGAGAGAGAGAAAACATTTCCCCATTTGCGACTACACCATTCAGGCAGTACAGAGAGCACACTAACACACAGTAGGAGAAAAAAAGAGGTGTG
TTCAACTGAGAAAAATTAATGGTGGCCATATATAAATACCACATATCAAAGAATGGGTAGAGCTCTGTTGAGTTTTCAGCTTGATAAATTTAGCA
GTTCCCTGTCTGCCATATTTGGGATACTGTAGTCTCTCTGTCCACAGGTTTCTACCTGAACAACCTCTAATTTGTGAGAAAGGAACTACCACTGTT
TCGCACCCAGCCCCGGAGCACGCACACTTCAGGCGTTATGGCCGCACCAACATTTGAAGGAGACACACCGAGAGAAGCCACATTCCTGCTATCA
GTTTGTCTTGCATCAAACAGCACCCCATTACTGAGGAGGGAGATAGAGCGTGTTCTCCTGAAGGAGGGAGAGTCTGCAGGAACCCATGATTGAA
TATTCACATTATGGCTAATTTGTTTTAGTTTTGGTTTTTGTTTTGTCTTCTAGGACAACAGTTATCATTCTTGATGATGAACATCAGTTCATGAGGA
GGTTATAAAAGGACAATAACGCGTTGATATAATAGGACAACAGTGATAATAATAATAATAAATAAAGAAATTTATATAGCGGCTTAATATGGTA
CTCTAAAGCTTAGTGATGATAATCTAATACATTGGACTAAAGAAGGTGTTGCTATTAACTGGTGATTATTATTATTTGGGAAAGAATAATTGAA

TAATATGGATATAGTAAAGATGAGAATAATAATTAAAAAAAAAAAAACGTAATCTTGATAAACTGTACAGAAGTAGAGTTCTTTTCTTCAAATGT
GGAATTCTTATCCTCTTTGGTCAAAGGATGAAATTTAATTAGGTACTAAAATTAAGGACTAATGCACTATGGCTGACGCCACCCCTTAATAAGGT
TGGCATCACATGAAAACTAACAGAACGACGCATCTAACCCGCATGAAATGCTTAAGGGTCGACCCATGCCTTCACTGCTTCGAAGGTCCCCCAC
AAAAGGGACTCCCATTATTGAAATAATTAGAAACTAGAGTTAAAAAGGTAAATATGTTTGAACATCTAAACTGTTGTACACCAGTTTGCTTAGTC
CAGCAAAGAGAAACACAAGTTCAAGGGCCGGAGGAGAACCCAGATGCAGGTACGGGCCGACCAACTACGAGATGATGCAATCAAATTTCGGTG
AGGTTTGAATCAGTCTGTTTTGGTGGTCCACTATTAACAAAAAGCTCGTTGGATAAATAATATATGGAGGCGGACCCCTACCACAAACTACAGA
GGTCGTCCAACCCAAACCCCTTGGCGTCACCCCAACACTGAAACATACACTTACACACACACACACAATCACGTCTGAACCAGGCTAAAAGATT
GTCTCTGCACATGGTGAAAGATGACCTGAATCAATCATTCAGTATGGATACCAGCCAGGACTGTGGTTTAGGCTGGGATTGATAGTGGTAGAAT
TTTCATTTTTCCAGGCGTGTGCGTGATTTTTGTGCTACCTTCCATAAAACGTGGGTATTGGCTAGATATCTGTTGCTACAGGCCATGGTTGGAAGG
ATAGGTGTGACCATTAGCATGCCTGTAGTTATTTAATAATTTTTTTTATTGGTGTTGACTTTCAATGCATGCTGAAAATTAGCATGAGACTTTGCG
TCTACATATATATTTAGAAGTTATTTGTTGTGTTTATGTTTCTTATTTTTTATGATTACACCTATATGTATTGCCCTTTGGATCTATAGTTAATGATT
TATTATTGTAATACTGATTATTGATTACGACTATTTTGGTTAATGATTTATTAACTTTACTACTGATTATTGATTTACTGTTTGGTTAATGATTTAT
TATTGATTTACTGGTACGGTTAATGATTTGTATTTCTTTACTACCGATTATTATTTATTGTTTTGTTGTGATTTATTATGATTTACTCTATGGTTAAT
GATTTATTAATAACACTACTGAATTATTGGTTACTGTTTGAGTTGTGATCTATTATTGCTTTACTACTGATTATTAGATTTTACTAATTTGATTATT
AATTTGAATATTATTAATTTCTGTGTATTCACCATAGTCTACTGTTGTATTGTTGACACTGGTCTGTATTTGAGACCCACCAGGTGAATTCGTTTC
AGGATTAGGGGGGGGCCGGTTGTGGTTTCACTCAATAAGGTGTGTTTTCCATAATGATTGGTGGTGTTGATGATTTTATACTTTTACACTTTGATA
CTTTTGTACCTTTTATACTTTTATACTTTTGTACACTTTTACATTTATACATTTATACATTTTATACTTTAGTACACCTTCTACTTTTATACCTTTATA
CATTTTATAAATTTTATACATTTTTCTATTTGCTTGTAGGATGGTCATCTGAGATGATCAAGGAGGAAATTATGTGTTCACACATTCATTGTATCC
TTACATATCTGCACAGGGACATATGGACTTTAATGTTGGATTCATTAGCATGGATTCATAATTTTTTTCAAACTTGTAATCCATGTAATGTACTTT
GATACATTATATAGTAGGAATTTCAAAGTATAGTCTTTGCCAGGTCTACTCTTCTTTCCAACAATGTGGTAGTAATG

>2d2665d1-fdcb-4189-9364-2efdfbeefe39 runid=9daf8b628adbbce9e9ad8e4177e9ecb123d0a169 read=21847 ch=511 start_time=2017-08-10T22:32:15Z
GAGGAAGAATTCCCCAGTCCAGCAAGAGACTAAAGGACATTCCCCAGTCAGACAGACAGAAGAACCTCGACCATGCCACAACTTTATTATGATT
AAAATTGGATACTGGATGATGAGAGAATGTAAGCCCGCCGAAAGATGGCCGTGACCGCTCCAAGCGAGAAGTGGGGGGCTCGAACAAAATGAA
ATTGAAGAGATTACAGGCCGAGTGTTGAAGAACTCAGCGCCTGTGCGATTCGAATGTTGAAACCAACAATAACACCAACACGGAATGATGACA
CTCGTTAGATTTGGCACGCAAACTAACTGATACGGGGTGAAAGAGTAGGAGTGTTTGAGTGTTTGTTATTTTCCTTTATTTTCACAAGAGAGAGT
TGTCTTCGAAAGATGCTACAGACTTGTCACTGCCTATCTAAGTTCCGTGTTGGTTCTTGACACAACAACCTGCTCAGCTTTAAATTTTTGTTTTGTT
TGTTTATTTTTGAACAAAGGTTCAATCCATTTCATTCATTCACACAAGGTTTATGGAGAACTACGATGACTGATTATGATTACTGAAGTATAACC
AGGCATTTTTGAATCAGGCGTTATTTTTTGTATGATAATTTTTTTCATTGAATCATTAAGACAATTACAAACCATAGTTGATTGACCAATACTGTT
GGTGACATGCCTCCCCATGACTGACTGCACTTTTTTTCTTTGTTTCATGTTTCATGCATTATCTGTCCAGGAGGCACTGTGGACGCAGTGAGTCTT
GAACTGATTTGTGTCTTGTGTTTGTCCTGACCTACTACGAACTTTCATCCTCATCCCCCATCACACCTGTGGTGGCCATCATGGAAACTCTGTTGC
AATTGCTACTCAAAACCAACTCTGGTGCAGTATTCTGTGATGTTGTGCCAAGCAGGGGTGTCTGGACGAGTTGACTTTCTTGATTCTTTAAACTTT
TTTTATGTTTGGTTTAATCATGAGCAACCTAATAGATTTTGTTATTTTCTTTATTGAAGTGGCCTACACCTGGTTTGCTGTGTATGATTTGAATTTA
CTTGTCCTATTGCCTTTTTATGCTTTCTTTCATGATTTGGATTAATTAGTAATGGATTATAGTAAATTACACTGAATTCTCATTTGGGTGATCTTTT
AAACTATGTTTAGTATAAGAGGGAGGAATGGCAGAATGTTGGTGTATTTTAATTGTCTCTTTTGTTCACATTTCTAGTCTAGGAACAGGCAAGAC
CTCTCATACCACTGATACAAAGGTGTTATCCAAACATTCTGCCATTGTTATGTTTCCAACGAGGTTGAACCGACCACAATGGGACATAGCTAGGG
CCAGATACCTTATCAAGGCTGAGAGTGCTGTTCGTGTTATGTATCCCCTTTTGTATAATATAGCCCCAACCCTTTGGTTTGAGTAGAGGTTCGACA
GCCAAGGAACAAGTCATCGACCGAGGTCCACTATAGATTATTCAACCTAGAGTGTATCTGCGTATTCCTGGAAAATCTGGACTGACCCTCTCTAA
TCCAACGAAGCTGCTTTGATTTGAGAATTACTACTGACTGAAAATACCTAGCGCAGGTCTGTCGAACGGTTTAAGAAATAAGCTGAAATCGCAG
CACACACATATAAACATCACCTCTTGCATACCACGCGCGCACAGAATCTCTCCAGACTTTCTCCTCCAAAGCATACACACATAAACATGCATACT
CTTATAGCGTACCCACACACAGAGATCTCTCAGACTTTTTTTCCTCAAAGCATACTTTATAAACATCTCTTTAACGTGCCTGATCTCTCGGCATCC
ATAGCAACCAGCGTTAAGCATTTACATAGCAAAAATGTGTGTCAACGCACCGCAGGGTTAGTCACTCAAAAAAAGAGATAATTACATTGGTCCA
AACCATATAATTCAGAACTGGCGCAATGTTGAGAACACTCTAGTAGAACATTATGGTCGAACACAGCTATATCGTGACATCTGAATCGAACAAT
ATGACTGGCACTTAATGAAGCCCAGACATACAAATTTGAACATTATAGACCGAACTGGCCTTCTCCGGCCGAGACGCCAGAATCCGAACATTAT
AGTGAACTGAAAGCACTTAATCGGCCCCAGACATAAAGTCGACATTATAATAATTCCCGAACGCTGGCATATATCCGGCCCGAGAACGCTCTGA
GATCCGAACATTATAGTGAGCGGCGCATGGCTCATTGAAGACATCCCTGAATCCGAACATTATAGTCAAGCAGCGCCAATCGACTGAGACGCCA
CAAGTCCGAACATTATGGTCGAACTGGGCAGTCGAACATTATATGGTTGAATTGGCGCTTGTCATTTGAGGCTGGAATCGAACATTATAGATGA
ACTGGCATATGTCCATTTGAACACTCTAAGAATCCGAACATTATAATTAAGTACTAGCATGCTCGGCCCAGTAAGAATCCGAGAAGATTATAAA
ACCAGTTGGCACTTCTCGGTCGAAACATGATCGAAGATTATAGTCGAACTGACACATAATCGACCCGGACACTCTGGACGAACATTAAAATTCG
AACTGGCATAATCATTGAGACATAATGTCGAACTGGCCATTCGGCCCGAGACATGGAATCGAACGATATGGTGAACGGCCATGGCGAGACATCT
AGTCAAATGATGAAGACGGCTATGTTGAGACATCTAAGTCCATTATAGTCGAACTGGCATAATGTTGACTGAGACATGATCCGAACTTTAAAAG
TCGAACTGGCATGTCGGCCCGAGACATACAAGTCGAACATTATAGTCGGAGCAGCATGTATATAACAACGCTCTTTTCTTATGGCATATGTAATA
ACATAACATCTTTAACAAAAGCATATCTCATATGCTCTCTTTCGCTCTCCCTCACGCACGCGGCATCGCTCACTCGTTTGCGCACTCTCGGTGGCG
TCGAACGTCATCTGGTGAGTGGAAAAACAAACGCACGCGCATGTCATAATAACGCTCTCGCACGCGCCTCTTTCTTTCTTTTTGCGCCTCTTTCTT
TTGCTGCGCTCGCCTCTTGCTCATCCGTAATATAATAATCAACAATATGCGCATCTAACGCGGCAAAAGCAATAATAATATCGTAACATCAACGT
AAATAATAATAATGATATCTCTCTATCAACGTAATATCAACGTAATCGAAATAAAGCATAATAATCGCGTGAAAATAAAAATGGCGTAATAACG
CGCTCATGTCGTATGGACATCGTGAAGATGACGGCGACAGCGGAACGACAATAATAGTGAAATAAACTCATCTTCGCCATCAATAATGGCGTAA
TAATATGTAACAGCAGCAATGAACAACATCATGAAAGACAAACAATATCGAAAATATGGCGCTCAATAAGGGGCAGACTGAAGGCGGCAAGCA
GCCTCCTGTGTGGCGTGAAGGTGACCAGCGTAGCAGCAACATGGCAGCGGCAGCCAGCCAGCAACAGCAGCGAAAGCGGCAGCAGCGGCAGC
AACGCGGCAATGGCATGGCAGCAGCGAAAACAGCGGCAGCAATGATGATGAAAAATGGCCTTGCCAGCGAAACTGAGCAGCAACAGCCTTCTT
GTAGCGGCAGCAGCAACCAGCGTAGCGGCAACGGTAAGCAGCCAGCAAGCAGCAGCAGCAACAGCAATATCAGCGGCAAAAGCGGCAGCAAT
GGCAGCGAAAGCCGTGGCAACGGCAACGACGGCGAAGCGAAAAGCGGCAGCGGCATCA

Supplementary file 4: The position of the eight autosomal gene copies of cod *zkY* in gadMor2.1 assembly.

Positions in GadMor2.1

**Gene**	**Linkage groups (scaffold)**	**Start**	**Stop**
zkY	LG11	12523752	12525260
zk1	LG17	19860627	19862524
zk2	GmG140706_s01175	13663	15561
zk3	LG08	5266991	5268680
zk4	LG12	832246	833025
zk5	LG03	56255	56716
zk6	LG04	15155501	15156349
zk7	LG07	4654270	4655625
zk8	LG22	446975	447911

Supplementary file 5: MAFFT alignments of cod zkY protein sequences and the eight autosomal copies in Atlantic cod.

```
CLUSTAL format alignment by MAFFT FFT-NS-i (v7.215)

zkY             ------------------------------------------------------------
zk1             MDPQTKRWPKESIWMVFKDWVKKTRRSEPVGLLLLSQCKRYFIKHVSNKKRDELEKTTAH
zk2             MDPQTKRWPKESIWMVFKDWVKKTRRSEPVGLLLLSQCKRYFIKHVSNKKRDELEKTTAH
zk3             ------------------------------------------------------------
zk4             ------------------------------------------------------------
zk5             ------------------------------------------------------------
zk6             ------------------------------------------------------------
zk7             ------------------------------------------------------------
zk8             ------------------------------------------------------------

zkY             ------MNWKRTRNKEQRKFTDLHTTLVREGLIAARPLDSG---GGDQGNGGGTLGVYGR
zk1             YEDTLGKTRELSFSPELTRERDLHQTLVREGLIAARPLDSGPPRGVDQGNGGGVLGIYGR
zk2             YEDTLGKTRELSFSPELTRERDLHQTLVREGLIAARPLDSGPPRGVDQGNGGGVLGIYGR
zk3             ----------------MRKLTDLHTTLARQGMIAASPLDSGPPRGCDQGNGGGTLGAYGR
zk4             ------------------------------------------------------------
zk5             ------------------------------------------------------------
zk6             ------------------------------------------------------------
zk7             ------------------MFTDLHTTLVGEGLIAAR-LDSGPPRGGDQGNGGGPLGVYGR
zk8             -----------------------------------------------------------M

zkY             MYPTLPSCGPEGEYIFPMQPSTPSGGLQESRHSVLSDVNSFIGEYPVICSTPHVPRKGNP
zk1             MYPTLPSCGPDGEYIFPMQPSSPPGGLQESRHSVLSAVNSFIGEYPVICSTPHVPRKGNP
zk2             MYPTLPSCGPDGEYIFPMQPSSPPGGLQESRHSVLSAVNSFIGEYPVICSTPHVPRKGNP
zk3             MYPTLPSCGPDGEYIFPMQPSLPSGGLQESRHSVLSDVNSFIGEYPVICSTPHVPRKGNP
zk4             ------------------------------------------------------------
zk5             ------------------------------------------------------------
zk6             ------------------------------------------------------------
zk7             MYPKLPLCGPDGEYIFP-------GGLQESRHS-----------DPVICSTPHVPRKGNP
zk8             ------------------------------------------------------------

zkY             SAPPDYANHDAIQIS---------------------------------------QQFQHP
zk1             SAPPDYANHDAIQIPQ----------QFPPVQHTLCVSQQFPQFSPVTPHSSIPQQFLHP
zk2             SAPPDYANHDAIQIPQ----------QFPPVQHTLCVSQQFPQFSPVTPHSSIPQQFLHP
zk3             SAPPDYANYDATQIPQQFPPQQFSPQQFPPVQHTLCVSQQFPQFSPVPPHSSLPQQFLHP
zk4             ------------------------------------------------------------
zk5             ------------------------------------------------------------
zk6             ------------------------------------------------------------
zk7             SAPPDYANHDAIQIP---------------------------------------------
zk8             ------------------------------------------------------------

zkY             NPPTQQPPEVRPGTVTKNESRIGGFDDNKQPHCSATGGAATPHMTTK-------------
zk1             NPPIQQPPEVRPGAVTKNESRIGGFDDNIQPHCSVMGGAITPHIKTTSKEEESEEEDKEG
zk2             NPPIQQPPEVRPGAVTKNESRIGGFDDNIQPHCSVMGGAITPHIKTTSKEEESEEEDKEG
zk3             HPPIQQPPEVRPGAVTKNESRIGGFDDNIQPHCSATGGAATPHIKTTSGEEEIDGEDREG
zk4             ------------------------------------------------------------
zk5             ------------------------------------------------------------
zk6             ------------------------------------------------------------
zk7             ----QQPPEVRPGRVTKNESRIGGFDDNKQPHCSVMGGAATPHMMTK-------------
zk8             ------------------------------------GGAATPNMKTT-------------

zkY             --------------------DEKDEIFNLRPLKKIKSAEGRTILYEPSSPKDIERWSAEI
zk1             ERLRKSAHKRAVSIACLNKVDEKDKIVNLRPLKKIESAEGRTILYEPSSPKNIEKWSADI
zk2             ERLRKSAHKRAVSIACLNKVDEKDEIVNLRPLKKIESAEGRTILYEPSSPKNIEKWSADI
zk3             ERLRKSAHKRAVSFACLKKVDEKDEIVNLGPLKKIESAEGRTILYEPSSPKDIERWSTEI
zk4             ------------------------------------------------------------
zk5             ------------------------------------------------------------
zk6             ------------------------------------------------------------
zk7             --------------------DEKDEIVNLRPLKKIESAEGRTILYEPSSPKDIERWSTEI
zk8             --------------------DEKDEIVSLRPLKKIESVEGRTILYEPSSPKDIEIWSAEI
```

```
zkY             EHPRRTGLQPWMKLKQLMLLYELHPYDGLAILFKHLSSNERTQIRYEVEDAIGEDGMRPG
zk1             EHPKRAGLQPWMELKQLMLLYELHPYDGLAILFKHLSSTERSQIRYEVEDALGEDGMRPE
zk2             EHPKRAGLQPWMELKQLMLLYELHPYDGLAILFKHLSSTERSQIRYEVEDALGEDGMRPE
zk3             DHPRRAGLQPWMKLKQLMLLYELHPYDGLAILFKHLSSTGRTQIRYEVEDAIGEDGMRPE
zk4             -----------MKLKQLMLLYELHPYDGLAILFKHLSSTERTQIRYDVEDAIGEDGMRPG
zk5             ------------------------------------------------------------
zk6             -----------MKLKQLMLLYELHPYDGLAILFKHLSSTERTQIRYEVEDALGEDGMRPE
zk7             DHRRRAGLQPWMKLKQLMLLYELHPYDGLAILFKHLSSTERTQIRYEVEDALGEDGMRPE
zk8             DHPRRAGLQPWMKLKQLMLLYELHPYDGLAILFKHLSGTERTQIRYDVEDAIGEDGMRPG

zkY             KGWEAINAWIQKHSRAQVNWTTITRCMQKEDEGLDQFNKRFFECYLLYSGQTEYDMDTID
zk1             KGWEAIKAWIQKHSKAQVNWTTITRCMQKEDEGLDQFNKRFFECYLLYSGQTEYDMDNID
zk2             KGWEAIKAWIQKHSKAQVNWTTITRCMQKEDEGLDQFNKRFFECYLLYSGQTEYDMDNID
zk3             KGWEAIKAWIQKHSKAQVNWTTITRCMQKEDEGLDQFNERFFECYLLHSGQTEYDMDTID
zk4             RGWGAIKAWIQKHSRAQVNWTTITRCMQKQDEGLDKFNERFFECYLLHSGQTEYDMDNID
zk5             --------------------------M---------------------------------
zk6             KGWEAIKAWIQKHSKAQVNWTTITRCLQKEDESLDQFNERFFECYLLHSGQTEYDMDTID
zk7             KGWEAIKAWIQKHSKAQVNGTTITRCMQKEDEGQDQFNERFFECYLLYSGQTEYDMDNID
zk8             KGWEAIKAWIQKHSRAQVNWTTITRCMQKEDEGLDQFNKRFFECYLLYSGQTEYDMDNID
                                          :

zkY             QSQDLPLKTMYLQQVLPEIRRGIKTRFPGWDNQSSTMAEIKELGEKVDRDEEVRIRFLAD
zk1             QSQDLPLKTIYLQQVLPEIRRGVKTRLPSWDNSKCTMAEIKEFGEKVDRDEEVRIRFLAD
zk2             QSQDLPLKTIYLQQVLPEIRRGVKTRLPSWDNSKCTMAEIKEFGEKVDRDEEVRIRFLAD
zk3             QSQDLPLKAMYLQQVLPEIRRGVKTRFPGWDNQSSTMAEIKEFGEKVDRDEEVRIRFLAD
zk4             RSQDLPLKTMYLQQVLPEIRRGVKTSLPSWDNGSCTMADIKEVGEKVERDEEVRIRFLAD
zk5             ----------YLQQVLPDIRRGVKTRLPSWDNSNCTMAEIKEFGEKVDRDEEVRIRFLAE
zk6             QSQDLPLKTMYLQQVLPEIRRGVKTRFPGWDNQSSTMAAIKEFGEKVDRDEEVRIRFLAD
zk7             QSQDLPLKTMYIQQVLPEIRRGVKTRLPGWDNQSSTMAAIKEFGEKVDRDEEVRIRFLAD
zk8             QSQDLPLKTMYLQQVLPEIRRGVKTRLPSWDNRSCTMAEIKEVGEKVDRDEEVRIRFLAD
                          *:*****:****:** :*.*** ..*** ***.****:***********:

zkY             EKKGMARERGRFPQSNRDRGRFPQSD----------RQKEPRPCHNCGQIGHWMRECKAP
zk1             EKKEIARERERFPQSNRDRGRFPQSD----------RQKEPRPCHNCGQIGHWMRECKAP
zk2             EKKEIARERERFPQSNRDRGRFPQSD----------RQKEPRPCHNCGQIGHWMRECKAP
zk3             EKKGMARERERFPQSNRDRERFPQPD----------KKKEPRPCHNCGQIGHWIRECKAP
zk4             EKKEIARDRERFPQSNRDRERFTQSD----------RRREPRPCHNCGQIGHWIRECKAP
zk5             EKKEMARERERFPQSNRDRGRFPQSD----------RQKEPRPCHNCGQIGHWIRECKTP
zk6             EKKEMARERERFPQSNRDRGRFPQSKRDRGRFPQPVRQKEPRPCHNCGQIGHWIRECKAP
zk7             EKKEMARERERFPQSNRDRGRFPQSNRDRGRFPQPVRQKEPRPCHNCGQIGHWIRECKAP
zk8             EKKEIARDRERFIQSD----------RQREPRPCHKCRQIGHWQRECKAP
                *** :**:* ********* ** *..          :::******:* ***** ****:*

zkY             RKMYRDRSNERVGEENRSDKMRERLKKLQAKSVEELSRLCDSVLETNNNTNLE
zk1             RKMYRDRSNERVGEENRSDKMRERLKKLQAKSVEELSRLCDSVLETNNNTNLE
zk2             RKMYRDRSNERVGEENRSDKMRERLKKLQAKSVEELSRLCDSVLETNNNTNLE
zk3             RKMHRERSNEKEGDENRSDKMKELWKKLQSQSAEELSRICNSMSETNN----K
zk4             RKMHRDRSNEKVWEENLSDEKRELLKKLQVKSVEELSRLCD------------
zk5             RKMYRDRSNERVGEENRSNKMRERLKKLQAKSVEELSRLCDSVLETNNNTNLE
zk6             RKMYRDRSNERVGEENRSDKMRERLKKLQAKSVEELSRLCDSVLETKNNTNLE
zk7             RKMYRDRSNERVGEENRSDKMRELLKKLQA-----------------------
zk8             RKMYRDRSNERVGEENRSDKMRELLKKLQA-----------------------
                ***:*:****:  :** *:: :*  ****
```

Supplementary file 6: MAFFT alignments of the Atlantic cod Y-sequence with Y- PCR product sequences of gadoids.

```
CLUSTAL format alignment by MAFFT (v7.402)


GadusMorhua_Y   AAAAGGAGGGGAATTGTACAAACACGTGTGGAGATACACTTAGTAGGGCATTCAAAAGGA
GadusOgac_Y     -------------------------------------TTTAGGTAGGGCATTC-AAAGGA
Arctogadus_Y    ------------------------------------------------CATTC-AAAGGA
                                                                ***** ******

GadusMorhua_Y   TGCTTTACCTGGAGCGTTCTAACCCCCTGGGCTATGTGTTGGGACTTATCTA-CTGGGCA
GadusOgac_Y     TGCTTTACCTGGAGCGTT--------CTGGGCTATGTGTTGGGACTTATCTANNNNNNNN
Arctogadus_Y    TGCTTTACCTGGAGCGTTCTAGCTCGCTGGGCTATGGGTTGTAA--NNNNNNNNNNNNNN
                ******************        ********** **** .*

GadusMorhua_Y   GATGTGGACTCAGTTATGTGCTCTGATTGGCTAAGCATG---------------------
GadusOgac_Y     NNNGTGGACTCAGTTATGTGCTCTGATTGGCTAAGCATGGTGCTGAACTCCCGCCTCTCT
Arctogadus_Y    GGCGTGGACTAAGTTATGTGCTCTGATTGGCTAAGCATGGTGCTGAACTCCCGCCTCTCT
                   ******* ****************************

GadusMorhua_Y   -----------GTGTCTCTATGCTGCTCCCTTGTGGCTATGTGTTGGCATTGCAATTTGT
GadusOgac_Y     GGATGTTGTATGTGTCTCTATGCTGCTCCCTTGTGGCTATGTGTTGGCATTGCAATTTGT
Arctogadus_Y    GGATGTTGTATGTGTCTCTATGCTGCTCCCTTGTGGCTATGTGTTGGCATCCCTTTTT--
                           ***************************************. *  ***

GadusMorhua_Y   TATGTGGCCCTGAATTTCTGAGTTCATCGAACACCTGGGCCGCACGTATCTGTAGAATTG
GadusOgac_Y     TATGTGGCCCTGAATTTCTGAGTTTGTCGAACACCTGGGCCGCACGTATCTGTAGAATTG
Arctogadus_Y    ------------------------------------------------------------


GadusMorhua_Y   ACACATGACGAATGGCCTCCTTTATGAGCTGGACGCCTCCCTAGGCTCCGGATCCCTCCT
GadusOgac_Y     ACACATGACAAATGGCCTCCTTTATGAACTGGACGCCTCCCTAGGCTCCGGATCCCTCCT
Arctogadus_Y    ------------------------------------------------------------


GadusMorhua_Y   TAGCGTGATAAAGAAGACTCTTTCATTGTTAAGGGCATATGTGGCTTTGGTATGAGTGTG
GadusOgac_Y     TAGCGTGATAAAGAAGACTCTTTAATTGTTAAGGGCATATGTGGCTTTGGTATGAGTGTG
Arctogadus_Y    ------------------------------------------------------------


GadusMorhua_Y   TGAATTACGTTACATGGCTATGTGCGGGAATTACACTTGTTCTTGTGGCCTTGAGCGTCT
GadusOgac_Y     TGAATTACGTTACATGGCTATGTGCGGGAATTACACTTGTTCTTGTGGCCTTGAGCGTCT
Arctogadus_Y    ------------------------------------------------------------


GadusMorhua_Y   GGGTCTCTAAAACATCCTGACCGCTCGTATCTCTAGAATTGACACATGATGGATGGCCTC
GadusOgac_Y     GGGTTTCTCGAACATCCTGACCGCTCGTATCTCTAGAATTGACACATGATGGATGGCCTC
Arctogadus_Y    ------------------------------------------------------------


GadusMorhua_Y   CTGTATGAGCTGGACGCCTCCCTAGGCTCCGGATCTCTCCTTAGCGTGATAAAGAAGACT
GadusOgac_Y     CTGTATGAGCTGGACGCCTCCCTAGGCTCCGGATCCCTCCTTAGCGTGATAAAGAAGACT
Arctogadus_Y    ------------------------------------------------------------


GadusMorhua_Y   CTTTAATTGGTAAAGGCATGTGTGGCCTTGGTATGAATGTGTGAATTACGTTACAGCACA
GadusOgac_Y     CTTTAATTGGTAAGGGCATATGTGGCCTTGGTATGAATGTGTGAATTACGTTACAGCACA
Arctogadus_Y    ------------------------------------------------------------


GadusMorhua_Y   CATTGTTTATTTATTTACACAGTGTTGTTTATTCAAAGGTTCGTATGTCTCCACCATTCC
GadusOgac_Y     CATTGTTTATTTATTTACACAGTGTTGTTTATTCAAAGTTTCGTATGTCTCCACCATTCC
```

```
Arctogadus_Y     ------------------------------------------------------------


GadusMorhua_Y    TGCTGACTTATAGGATTGATTGCAGCTGATCAATGTGAGTCAATGCGTACCAGTGCCCCA
GadusOgac_Y      TGCTGACTTATAGGATTGATTGCAGCTGATCAATGTGAGTCAATGCGTAGCAGTACCCCA
Arctogadus_Y     ------------------------------------------------------------


GadusMorhua_Y    TTAGCTTGGCATCCATTT-GGCACACTTTTAGATACCCAGCGGAAGAAAATATG
GadusOgac_Y      TTAGCTTGGCATCCATTTTGGCACAC----------------------------
Arctogadus_Y     ------------------------------------------------------
```

Supplementary file 7: MAFFT alignments of the Atlantic cod X-sequence with X- PCR product sequences of gadoids.

```
CLUSTAL format alignment by MAFFT (v7.402)


GadusMorhua_X   -TCACATTGATCAGCTGC-ATCAATCCTATAAGTCAGCAGGAAT-GGTGGAGACATACGA
GadusOgac_X     CTCACATTGATCAGCTGC-ATCAATACTATAAATCAGCAGGAAT-GGTGGAGACATACGA
Arctogadus_X    ------TTGATCAGCTGC-ATCAATCCTATAAGTCAGCAGGAAT-GGTGGAGACATACGA
Boreogadus_X    CTCACATTGATCAGCTGC-ATCAATCCAATAAGTCAGCAGGAAT-GGTGGAGACATACGA
Melanogrammus_X ----CATTGATCAGCTACAATCAATCCTATAGGTCAGCAGGAAT-GGTGGGGACATACGA
LotaLota_X      ------TTGATCAGCTGC-ATCAATCCTCTAGGTCAGCAGGAATGGGTGGAGACATATGN
                      **********.* ****** *  **..*********** *****.******.*

GadusMorhua_X   ACCTTTGAATAAACAACACTGTGTAAACAAATAAATAATGTGTGCCAATGCGGTTGTTAG
GadusOgac_X     ACCTTTGAATAAACAACACTGTGTAAATAAATAAATAATGTGTGCCAATGCAGTTGTTAG
Arctogadus_X    ACCTTTGAATAAACAACACTGTGTAAATAAATAAATAATGTGTGCCAATGCAGTTGTTAG
Boreogadus_X    ACCTTTGAATAAACAACACTGTGTAAATAAATAAATAATGTGTGCCAATGCAGTTGTTAG
Melanogrammus_X ACCTTTGAATAAACAACACTGTGTAAATAAATAAATAATGTGTGCCAATGCAGTTGTTAG
LotaLota_X      NNNNNNNNNNTCATCGAA---TAAAAACACATAAATAATGTGTGTCAACGCAGTTGTTAG
                            *. . *   *. ***.* *************.***.**.********

GadusMorhua_X   TGCCTCTAAAAGCAACCAATGTGACCTCATCAATGTTTGCACAAAAGTTTCATAGTTATT
GadusOgac_X     CGCCTCTAAAAGCAACCAATGTGACCTCATCAATGTTTGCACAAAAGTTTCATAGTTATT
Arctogadus_X    CGCCTCTAAAAGCAACCAATGTGACCTCATCAATGTTTGCACAAAAGTTTCATAGTTATT
Boreogadus_X    CGCCTCTAAAAGCAACCAATGTTACCTCATCAATGTTTGCACAAAAGTTTCATAGTTATT
Melanogrammus_X CGCCTCTAAAAGCAACCAATGTTACCTCATCAATGTTTGCACAAAGGTTTCATAGTTATT
LotaLota_X      TGCCTCTAAAAGCA---------ACCTCACCAGTGTTTGTATAAAGGTTTCACAGTTGTT
                .*************         ******.**.******.*.***.******.****.**

GadusMorhua_X   AGTGCTGCGAGTACAAAATGCATTGACATTGTGATCCAATATAAT-ACACAATTTATAAA
GadusOgac_X     AGTGCTGCGAGTACAAAATGCATTGACATTGTGATCCAATATAAT-ACGCAATTTATAAA
Arctogadus_X    AGTGCTACGAGTACAAAATGCATTGACATTGTGATCCGATATAAT-ACGCAATTTATAAA
Boreogadus_X    AGTGCTACGAGTACAAAATGCATTGACATTGTGATCCAATATAAT-ACGCAATTTATCAA
Melanogrammus_X AGTGCTACGAGTAAAAAAGGCATTGGCATTGTGATCCAATATAATAACGCAATTTATAAA
LotaLota_X      TGTGCTACGAGTACAACATTTATTGACATTGTGAACCAATATAAT-ACGTAATTTTTAAT
                 *****.****** ** *  .****.******** **.******* **..***** * *

GadusMorhua_X   TCACCTCTTTAATAAGAGCAATTTTCTTTTCCTATTATAATATTTTGA------------
GadusOgac_X     TCACCTCTTTAATAAGAGCAATTTTCTTTTCCTATTATAATATTTTGA------------
Arctogadus_X    TCACCTCTTTAAATAGAGCAATTTTCTTTTCCTATTATAATATTTTGA------------
Boreogadus_X    TCACCTCTTTAATTAGAGCAATTTTCTTTTCCTATTATAATATTTTGA------------
Melanogrammus_X TCACCTCTTTAATTAGAGCAATTTTCTTTTCCTATTATAATATTCTGA------------
LotaLota_X      TCACCTCTTTAATTAGAGCAACTTTATATTTTTATTATAATGTTTTGAACTTTGGTGCTG
                ************  *******.*** * **..*********.**.***

GadusMorhua_X   ACTTTCCAAATGAATAATTATGAATCATTGGGCAGCAGAAATTATTAATAA--TCAGAGT
GadusOgac_X     ACTTTCCAAATGAATAATTATGAATCATTGGGCAGCAGAAATTATTAATAA--TCAGAGT
Arctogadus_X    ACTTTCCAAATGAATAATTATGAATCATTGGGCAGCAGAAATTATTAATAA--TCAGAGT
Boreogadus_X    ACTTTCCAAATGAATAATTATGAATCATTGGGCAGCAGAAATTATTAATAA--CCAGAGT
Melanogrammus_X ACTTTCCAAATGAATAATTCTGAATCATTGGGCAGCAGAAATTATTAATAA--TCAGAGT
LotaLota_X      ACTTTCAAAAGGAATAATAATGAATCACTGGGCTGCAGGAATGATTATTAATTTCAGAGT
                ****** *** *******  *******.***** ****.*** **** ***   .******

GadusMorhua_X   TTGCATGTTA----TATGTTACT-CTGAATACCGCTCTTCAAAGCACAGTGGGATGGCTT
GadusOgac_X     TTGCATGTTA----TATGTTACT-CTGAATACCACTCTTCAAAGCACAGTGGGATGGCTT
Arctogadus_X    TTGCATGTTA----TATGTTACT-CTGAATACCGCTCTTCAATGCACAGTGGGATGGCTT
Boreogadus_X    TTGCATGTTA----TATGTTACT-CTGAATACCGCTCTTCAATGCACAGTGGGATGGCTT
Melanogrammus_X TTGCATGTTA----TATGTTACT-CTGAATACCGCTCTTCAAAGCACAGTGGGATGGCTT
LotaLota_X      TTGCATGTTATAAGTATGTTACTCCTGAATATCGCTCTTCAAAGCACAACGGGATGGCTT
                **********    ********* *******.*.******** *****..**********

GadusMorhua_X   ATGTAAAAGGTTAGCTCAGCCCGCGAATAATTAAAATAGAT-GGGTAGAATGAACACGTT
```

```
GadusOgac_X     ATGTAAAAGGTTAGCTCAGCCCGCGAATAATTAAAATAGAT-GGGTAGAATGAACACGTT
Arctogadus_X    ATGTAAAAGGTTAGCTCAGCCCGCGAATAATTAAAATAGAT-GGGTAGAATGAACACGTT
Boreogadus_X    ATGTAAAAGGTTAGCTCAGCCTGTGAATAATTAAAATAGAT-GGGTAGAATGAACACGTT
Melanogrammus_X ATGTAAAAGGTTAGCTCAGCCCGCGAATAATTAAAATCGAT-GGGTAGAATGAACACGTT
LotaLota_X      CTGTAAAAGGTTAGCTCAGCCCCAGAATAATTCAAATAGACAGGGAAGAATGAACACGCA
                 ********************.  ******** **** **. *** ************.

GadusMorhua_X   AAGTTATGCCAAAGTCTTGAAATATGTGCCGATTGCCACATTGATTACACTGTCCCGAAC
GadusOgac_X     AAGTTATGCCAAAGTCTTGAAATATGTGCCGATTACCACATTGATTACACTGTCCCGAAC
Arctogadus_X    AAGTTATGCAAAAGTCTTGAAATATGTGCCGATTACCACATTGATTACACTGTCCCGAAC
Boreogadus_X    AAGTTATGCCAAAGTCTTGAAATATGTGCCGATTACCACATTGATTACACTGTCCCGAAC
Melanogrammus_X AAGTTATGCAAAAGTCTTGAAATATGTGCCGATTACCACATTGATTACACAGTCTCAAAC
LotaLota_X      AAGTTGAGCAAAAATCTTGAAATACGAGCAGATTACCACATAGATTAGATTATTACGTAA
                *****. ** ***.**********.* ** ****.****** ***** *. .*. *. *

GadusMorhua_X   AACTATAAGGGCATATCTGCACAGGGACATATATGGACTTTACTGTTGGATTCGTAGC--
GadusOgac_X     AACTATAAGGGCATATCTGCACAGGGACATATATGGACTTTACTGTTGGATTCGTAGC--
Arctogadus_X    AACTATAAGGGCATATCTGCACAGGGACATATATGGACTTTAATGTTGGATTCGTAGCAT
Boreogadus_X    AACTATAAGGGCATATCTGCACAGGGACATATATGGACTTTAATGTTGGATTCGTAGCAT
Melanogrammus_X GACTATATGGGCATATCTGCACAGGGACATGTATGGACTTTAATGTTGCATTCATAGCAT
LotaLota_X      GACTACACGGGCACATCTGCACGGGGATATATATGGAC-----TGTTTGATTCATAGCAT
                .****.* *****.********.****.**.*******     ****  ****.****

GadusMorhua_X   --ATTCATAAT-TTTTTTCAAGCAATAATCCATACTGTGGCACGCATTAAATAATATCAT
GadusOgac_X     --ATTCATAAT-TTTTTTCAAGCAATAATCCATACTGTGGCACGCATTAAATAATATCAT
Arctogadus_X    GGATTCATAAT-TTTTTTCAAGCAATAATCCATACTGTGGCACGCATTAAATAATATCAT
Boreogadus_X    GGATTCATAAT-TTTTTTCAAGCAATAATCCATACTGTGGCACGCATTAAATAATATCAT
Melanogrammus_X GGATTCATAAT-TTTTTTCAAGCAATAATCTATACTGTGGCACGCATTAAATAATATCAT
LotaLota_X      GGATTCATCATGTTTTATCAAGCAATAATCCATACAGTGTAACGCGCTACATATCATATA
                  ****** ** **** *************.**** ***  ****..** *** .**

GadusMorhua_X   AGAATTTCAAAGTATAGTCTTTGCAAGTCTATAG-CTAACAACACCAACAATGTGAGTAG
GadusOgac_X     AGAATTTCAAAGTATAGTCTTTGCAAGTCTATAG-CTAACAACACCAACAATGTGAGTAG
Arctogadus_X    AGAATTTCAAAGTATAGTCTTTGCAAGTCTATAG-CTAACAACACCAACAATGTGAGTAG
Boreogadus_X    AGAATTTCAAAGTATAGTCTTTGCAAGTCTATAG-CTGACAACACCAACAATGTGAGTAG
Melanogrammus_X ATAAGAACCAAGCCCGATTTTT----TTTTATAGCCTGACACGAACATGGATTTATCTGT
LotaLota_X      AGAACCCAAG-----------CCTGATTGTATCC-CGGATATAACCATAAGTGTGTCCAT
                * **     .           .     * ***   * .*.*  * **  ..* *.  ..

GadusMorhua_X   TAGCCTGAGAAAAGAGGACCT-GGTTTCAGAGCATTAATTTAGATCTGTGTCAAGGCTGG
GadusOgac_X     TAGCCTGAGAAA------------------------------------------------
Arctogadus_X    TAGCCTGAGAAAAGAGGACCTGGGTTTCAGAGCATTAATTTAGAACTGTGTCAAGGCTGG
Boreogadus_X    TAGCCTGAGAAAAGAGGACCTGGGTTTCAGAGCATTAATTTAGAACTGTGTCAAGGCTGG
Melanogrammus_X TAATATTAG---------TCTTCATTTCAAAATGCCAAT---------------------
LotaLota_X      AAAACCTGA-AACGTGCTTCTGTGCATCTGGGCGTGCATACA------------------
                 *.  . ..

GadusMorhua_X   GCTTAAACTACGATGTTATCGTTTTGCGGTTAACTACGGAT-
GadusOgac_X     ------------------------------------------
Arctogadus_X    GCTTAAACTACGATGTTATCGTTTTGCGGTTAACTACGGATC
Boreogadus_X    GCTT-AACTACGATGT-ATCGTTTTGC---------------
Melanogrammus_X -----AATTCAAACATAGGTGTTTT-----------------
LotaLota_X      -------------TGTAGACATT-------------------
```

Supplementary file 8: 102 samples ids, sex and ENA accession id.

Sample ID	Sex	ENA Study ID	Secondary accession
Sample_1	Female	PRJEB12803	ERP014312
Sample_105	Female	PRJEB12803	ERP014312
Sample_108	Male	PRJEB12803	ERP014312
Sample_13	Female	PRJEB12803	ERP014312
Sample_132	Female	PRJEB12803	ERP014312
Sample_133	Female	PRJEB12803	ERP014312
Sample_134	Male	PRJEB12803	ERP014312
Sample_138	Female	PRJEB12803	ERP014312
Sample_140	Male	PRJEB12803	ERP014312
Sample_15	Male	PRJEB12803	ERP014312
Sample_151	Female	PRJEB12803	ERP014312
Sample_152	Male	PRJEB12803	ERP014312
Sample_153	Female	PRJEB12803	ERP014312
Sample_155	Male	PRJEB12803	ERP014312
Sample_162	Female	PRJEB12803	ERP014312
Sample_164	Male	PRJEB12803	ERP014312
Sample_168	Female	PRJEB12803	ERP014312
Sample_172	Female	PRJEB12803	ERP014312
Sample_173	Male	PRJEB12803	ERP014312
Sample_199	Female	PRJEB12803	ERP014312
Sample_200	Male	PRJEB12803	ERP014312
Sample_201	Female	PRJEB12803	ERP014312
Sample_205	Male	PRJEB12803	ERP014312
Sample_214	Female	PRJEB12803	ERP014312
Sample_215	Male	PRJEB12803	ERP014312
Sample_223	Female	PRJEB12803	ERP014312
Sample_224	Male	PRJEB12803	ERP014312
Sample_230	Male	PRJEB12803	ERP014312
Sample_249	Female	PRJEB12803	ERP014312
Sample_251	Male	PRJEB12803	ERP014312
Sample_253	Female	PRJEB12803	ERP014312
Sample_256	Female	PRJEB12803	ERP014312
Sample_258	Male	PRJEB12803	ERP014312
Sample_267	Male	PRJEB12803	ERP014312
Sample_27	Female	PRJEB12803	ERP014312
Sample_271	Female	PRJEB12803	ERP014312
Sample_272	Female	PRJEB12803	ERP014312
Sample_274	Female	PRJEB12803	ERP014312
Sample_276	Male	PRJEB12803	ERP014312

Sample_28	Male	PRJEB12803	ERP014312
Sample_280	Female	PRJEB12803	ERP014312
Sample_283	Female	PRJEB12803	ERP014312
Sample_285	Male	PRJEB12803	ERP014312
Sample_286	Female	PRJEB12803	ERP014312
Sample_291	Male	PRJEB12803	ERP014312
Sample_3	Male	PRJEB12803	ERP014312
Sample_303	Male	PRJEB12803	ERP014312
Sample_304	Female	PRJEB12803	ERP014312
Sample_310	Female	PRJEB12803	ERP014312
Sample_312	Male	PRJEB12803	ERP014312
Sample_328	Female	PRJEB12803	ERP014312
Sample_329	Male	PRJEB12803	ERP014312
Sample_33	Female	PRJEB12803	ERP014312
Sample_338	Male	PRJEB12803	ERP014312
Sample_347	Male	PRJEB12803	ERP014312
Sample_349	Female	PRJEB12803	ERP014312
Sample_35	Male	PRJEB12803	ERP014312
Sample_350	Male	PRJEB12803	ERP014312
Sample_36	Female	PRJEB12803	ERP014312
Sample_363	Female	PRJEB12803	ERP014312
Sample_364	Female	PRJEB12803	ERP014312
Sample_365	Male	PRJEB12803	ERP014312
Sample_37	Male	PRJEB12803	ERP014312
Sample_387	Female	PRJEB12803	ERP014312
Sample_389	Male	PRJEB12803	ERP014312
Sample_40	Male	PRJEB12803	ERP014312
Sample_400	Female	PRJEB12803	ERP014312
Sample_402	Male	PRJEB12803	ERP014312
Sample_403	Female	PRJEB12803	ERP014312
Sample_405	Male	PRJEB12803	ERP014312
Sample_408	Male	PRJEB12803	ERP014312
Sample_41	Female	PRJEB12803	ERP014312
Sample_422	Female	PRJEB12803	ERP014312
Sample_424	Male	PRJEB12803	ERP014312
Sample_43	Male	PRJEB12803	ERP014312
Sample_433	Male	PRJEB12803	ERP014312
Sample_436	Female	PRJEB12803	ERP014312
Sample_437	Male	PRJEB12803	ERP014312
Sample_448	Female	PRJEB12803	ERP014312
Sample_449	Male	PRJEB12803	ERP014312
Sample_456	Female	PRJEB12803	ERP014312
Sample_458	Male	PRJEB12803	ERP014312
Sample_46	Female	PRJEB12803	ERP014312
Sample_47	Male	PRJEB12803	ERP014312

Sample_477	Female	PRJEB12803	ERP014312
Sample_494	Female	PRJEB12803	ERP014312
Sample_496	Male	PRJEB12803	ERP014312
Sample_497	Female	PRJEB12803	ERP014312
Sample_510	Female	PRJEB12803	ERP014312
Sample_511	Male	PRJEB12803	ERP014312
Sample_63	Female	PRJEB12803	ERP014312
Sample_69	Female	PRJEB12803	ERP014312
Sample_72	Male	PRJEB12803	ERP014312
Sample_73	Female	PRJEB12803	ERP014312
Sample_75	Male	PRJEB12803	ERP014312
Sample_79	Female	PRJEB12803	ERP014312
Sample_80	Male	PRJEB12803	ERP014312
Sample_84	Female	PRJEB12803	ERP014312
Sample_85	Female	PRJEB12803	ERP014312
Sample_87	Male	PRJEB12803	ERP014312
Sample_88	Female	PRJEB12803	ERP014312
Sample_89	Male	PRJEB12803	ERP014312

Supplementary figure 1: Schematic representation of *zkY* genomic region and synteny comparison across various teleosts and spotted gar. Gene order, chromosome/scaffold id and picture style was adapted from Genomicus (http://www.genomicus.biologie.ens.fr/genomicus-93.01/cgi-bin/search.pl). Genes are depicted by colored pentagon and transcriptional orientation is indicated at the angled end of each gene. Gene names are indicated above the first pentagons and ortholog genes across species are depicted in the same color.

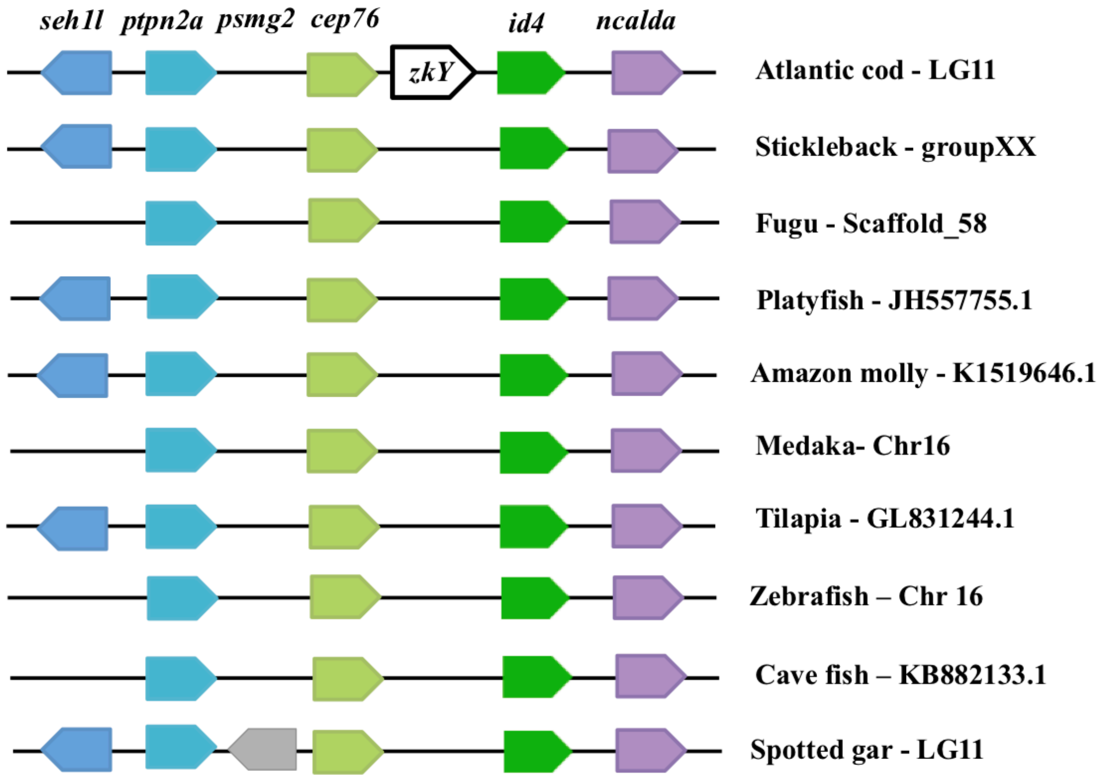

Supplementary figure 2: Phylogenetic tree based on amino acid sequences indicating the relationship of the zkY protein and its autosomal protein copies in a region surrounding the zinc knuckle domain. The phylogenetic tree was generated using MEGA 7.0.26 [1] maximum likelihood statistical method. Significant differences between the zkY transcript copies meant that it was only possible to compare a 248 amino-acid sequence for a region flanking the zinc knuckle domain (in zkY; position 231 to 479, see also supplementary file 5). The zk5 transcript was excluded from tree-building because it was relatively very short and to have included it in an isoform comparison, all other isoforms would have had to be trimmed to a similar length reducing the tree's analytical power.

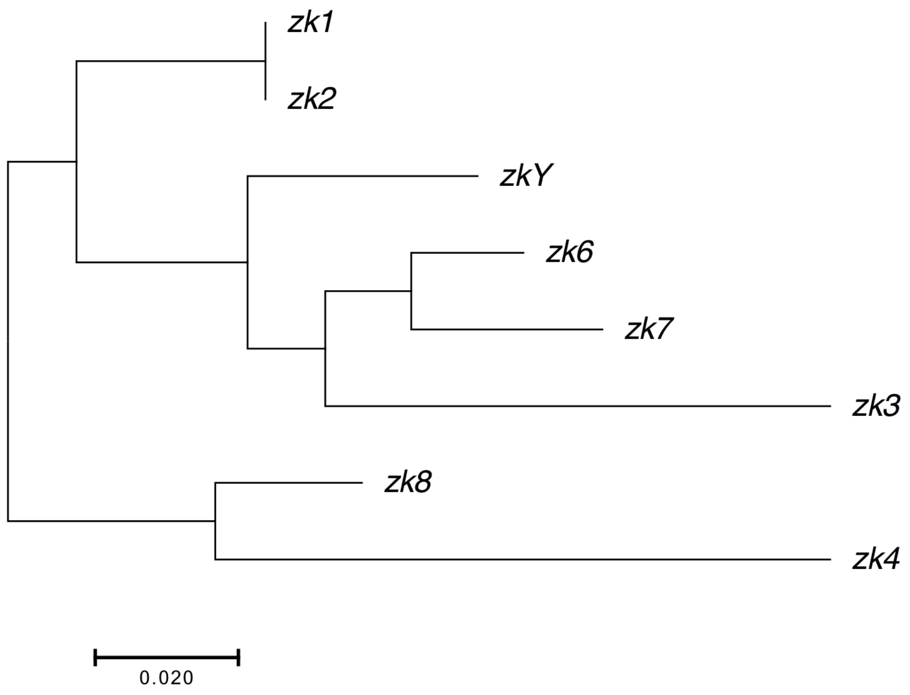

Supplementary figure 3: Time-calibrated phylogeny of Gadidae family, showing the presence of X- and Y- specific sequences as determined by PCR. Red circles and blue squares indicate the presence of X- and Y- PCR products respectively (See supplementary files 6 and file 7). The age estimates of species were taken from Malmstrøm *et al.* [2], except for *Gadus macrocephalus ogac* [3,4]. Species whose names are written in light grey were not analyzed with PCR.

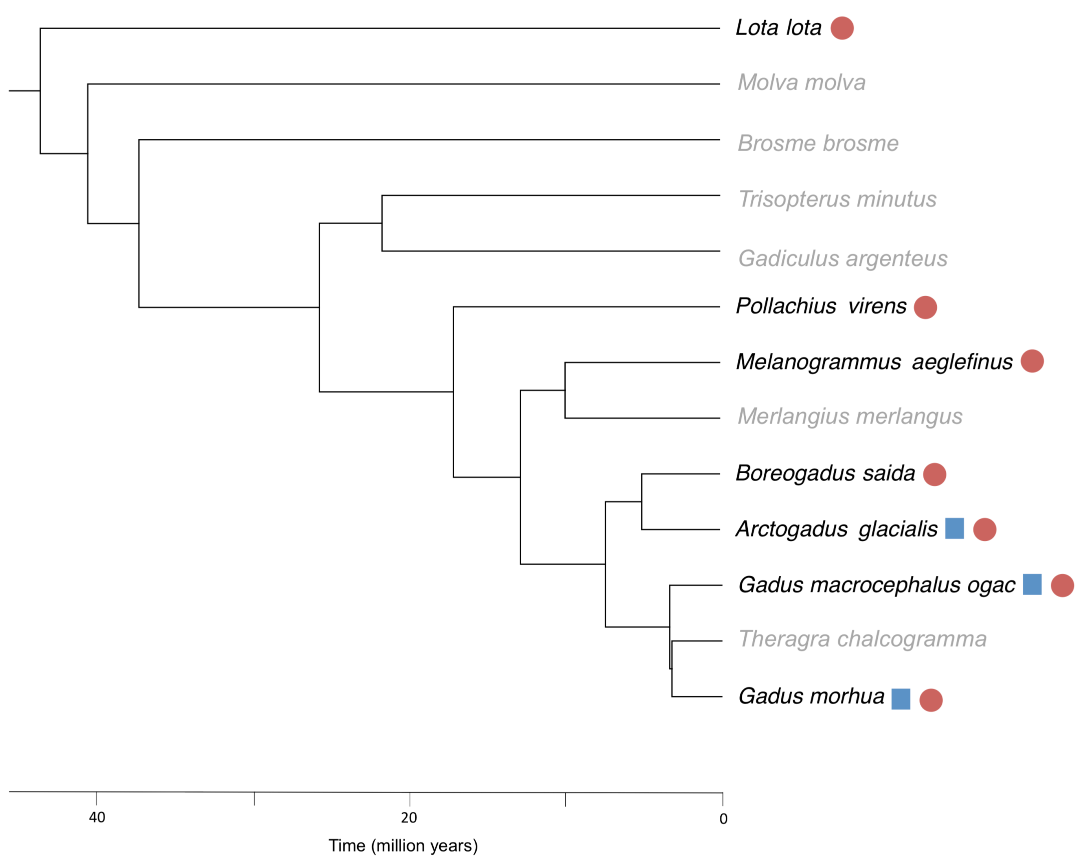

Supplementary figure 4: Full-length duplex PCR gel image designed for distinguishing sex in Atlantic cod. For the manuscript, we cropped the above gel image to include only the lanes highlighted inside the blue box, and flipped the image horizontally so that the ladder is on the left. Slight adjustments were made to improve contrast. The negative control lane contains product from a PCR reaction without DNA template. The image was captured using a BioRad GelDoc XR+, and image adjustment done using Image Lab Software V3.11.

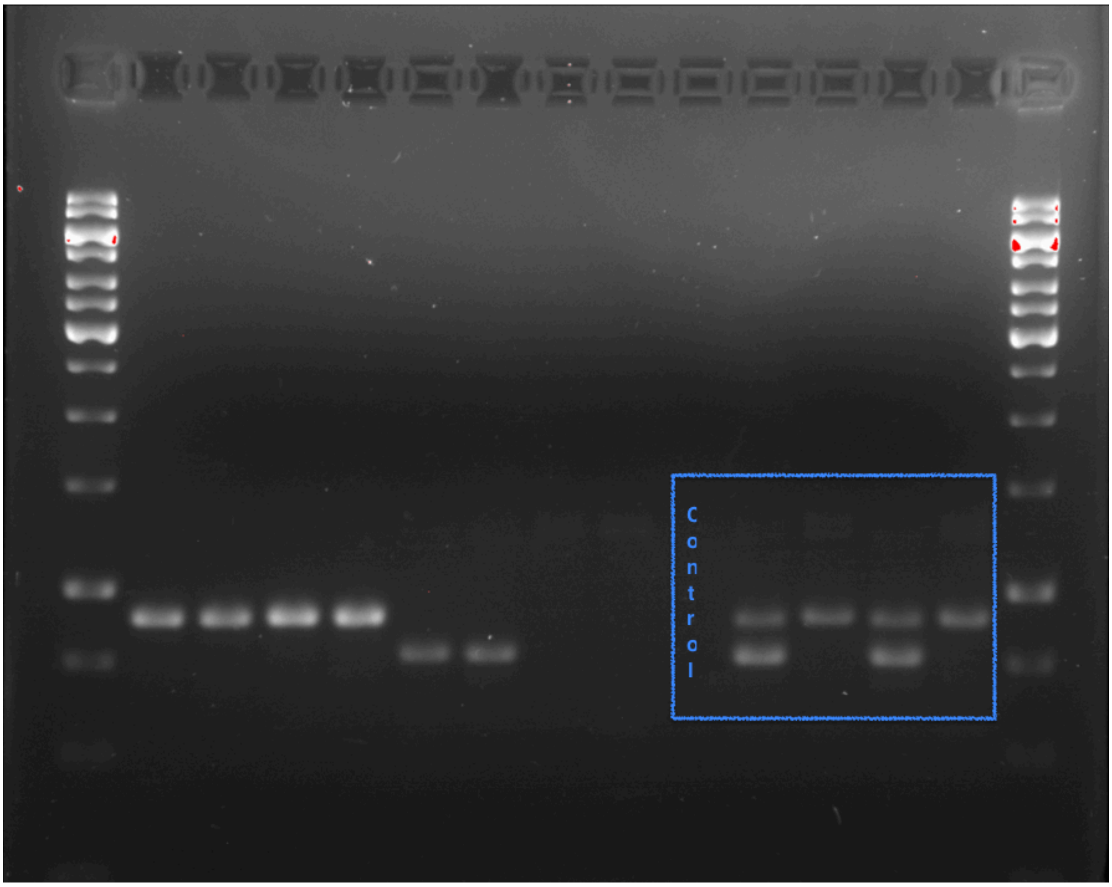

References:

1 Kumar, S., Stecher, G. & Tamura, K. MEGA7: Molecular Evolutionary Genetics Analysis Version 7.0 for Bigger Datasets. *Mol Biol Evol* **33**, 1870-1874,(2016).

2 Malmstrom, M. *et al.* Evolution of the immune system influences speciation rates in teleost fishes. *Nat Genet* **48**, 1204-1210,(2016).

3 Carr, S. M., Kivlichan, D. S., Pepin, P. & Crutcher, D. C. Molecular systematics of gadid fishes: implications for the biogeographic origins of Pacific species. *Can J Zool* **77**, 19-26,(1999).

4 Coulson, M. W., Marshall, H. D., Pepin, P. & Carr, S. M. Mitochondrial genomics of gadine fishes: implications for taxonomy and biogeographic origins from whole-genome data sets. *Genome* **49**, 1115-1130,(2006).
